# Genomic resources of Cold-adapted *Mrakia* yeasts and their potential biotechnological applications

**DOI:** 10.1038/s41598-025-29037-8

**Published:** 2025-12-22

**Authors:** Seung Chul Shin, Dieu Linh Nguyen, ChanSu Jeong, Yung Mi Lee, Jun Hyuck Lee, Se Jong Han, Han-Woo Kim

**Affiliations:** 1 Division of Life Sciences, Korea Polar Research Institute (KOPRI), Incheon, 21990 Republic of Korea; 2 Department of Polar Sciences, University of Science and Technology, Incheon, 21990 Republic of Korea; 3 Department of Pathology and Genomic Medicine, Houston Methodist Hospital, Houston, TX USA

**Keywords:** Biotechnology, Computational biology and bioinformatics, Genetics, Microbiology

## Abstract

*Mrakia* is a cold-adapted yeast genus commonly found in polar environments, including the Antarctic and Arctic regions. Several *Mrakia* genome sequences have been reported to date; however, comprehensive genomic analyses remain limited. Further, there are no reports of complete mitochondrial genome sequences for the genus *Mrakia*. Therefore, we isolated two cold-adapted yeast strains, *Mrakia gelida* PAMC 26583 and *Mrakia robertii* PAMC 26600, from Arctic lichens. Phylogenetic analysis placed both strains within the order Cystofilobasidiales. High-quality whole-genome assemblies were generated using Nanopore long-read and Illumina short-read sequencing, resulting in nearly complete nuclear and mitochondrial genomes. Analyses of gene family expansion and contraction revealed lineage-specific enrichment in functions associated with nucleosome assembly, DNA repair, metal ion binding, and membrane transport. Functional annotation identified numerous carbohydrate-active enzymes and peptidases with potential industrial applications. Both strains also exhibited ethanol production under low dissolved oxygen conditions, underscoring their utility in low-temperature fermentation. Notably, the mitochondrial genome annotation presented here represents the first reported for the order Cystofilobasidiales, providing valuable taxonomic insights into the class Tremellomycetes. These genomic resources will support future evolutionary studies and the development of biotechnological applications involving cold-active enzymes and low-temperature fermentation.

## Introduction

Cold-adapted (psychrophilic and psychrotolerant) yeasts play essential ecological roles in polar and alpine ecosystems and have gained attention for their ability to survive and remain metabolically active at low temperatures^1–3^. These organisms produce cold-active enzymes and metabolites that not only facilitate adaptation to extreme environments but also hold considerable potential for industrial applications, including food processing, biofuel production, bioremediation, and pharmaceutical development^4^.

Among cold-adapted yeasts, species of the genus *Mrakia* (Basidiomycota, Tremellomycetes, order Cystofilobasidiales) are frequently isolated from cold environments such as Arctic and Antarctic soils, glacial meltwaters, permafrost, and alpine habitats^5–8^. *Mrakia* spp. are true psychrophiles, characterized by their inability to grow above 20 °C and their preference for temperatures below 15 °C. They contribute to carbon cycling in these ecosystems by decomposing a wide range of organic materials, including remnants of cell walls and membranes, via cold-active enzymes^1^. In addition to their ecological importance, *Mrakia* spp. exhibit biotechnological potential, such as the production of cold-active lipases and proteases^2^, the ability to bioremediate^9,10^, and alcohol fermentation under low-temperature conditions^8,11,12^.

Given their ecological and industrial importance, several *Mrakia* genome sequences have been reported to date, including those of *M. gelida*^13^, *M. hoshinonis*^14^, and *M. blollopis*^15^; however, comprehensive genomic analyses—including gene annotation, comparative genomics, and functional profiling—remain limited. Additionally, to date, there have been no reports of complete mitochondrial genome sequences for the genus *Mrakia*.

In this study, we report the isolation and genome sequencing of two cold-adapted *Mrakia* strains, PAMC 26583 and PAMC 26600, from the Arctic region. We performed high-quality genome assemblies via a combination of Oxford Nanopore and Illumina sequencing technologies, followed by comprehensive comparative and phylogenetic analyses, as well as functional gene annotation. ITS and D1/D2 region analyses identified PAMC 26583 as *M. gelida* and PAMC 26600 as *M. robertii*, and phylogenetic positions within the genus *Mrakia* were confirmed. Gene family expansion and contraction were also examined. To evaluate their biotechnological potential, we analyzed the repertoire of carbohydrate-active enzymes (CAZymes) and peptidases. Notably, we report the first mitochondrial genome sequences for the genus *Mrakia*. Finally, we assessed the ethanol production capabilities of both strains under low-temperature, low-oxygen conditions. This study provides valuable genomic resources for cold-adapted yeasts and enhances our understanding of the genetic basis of their psychrophily and the industrial potential of *Mrakia* species.

## Results

### Strain isolation

The PAMC 26583 and PAMC 26600 strains were isolated from lichen samples (*Cladonia pyxidata* and *Cetraria* sp., respectively) in Arctic regions and were initially classified as *Mrakia* by analyzing sequences of the ITS region. PAMC 26583 was most closely related to *M. gelida*, with a sequence identity of 99.84% with *M. gelida* CBS 5272^T,^ and PAMC 26600 was most closely related to *M. robertti*, with a sequence identity of 99.8% with *M. robertii* CBS 8912 ^T^ in the ITS region. PAMC 26583 was isolated on Reasoner’s 2 A (R2A) agar plates at 10 °C for 10 days, whereas PAMC 26600 was cultured on Malt-Yeast (MY) agar plates at 10 °C for 10 days. The colony morphologies of the *Mrakia* strains PAMC 26583 and PAMC 26600 were assessed after 10 days of incubation on MY agar at 10 °C. Both strains produced circular, convex, entire, and glistening colonies (Table 1 and Supplementary Figure S1). To test the optimum temperature, both strains were cultured on 0.1× nutrient agar (NA) and MY agar plates for 7 days. At temperatures below 10 °C, both strains exhibited faster growth than at temperatures above 15 °C, with optimal growth observed between 4 °C and 10 °C, and no growth above 25 °C.

**Table:** Table 1.

Isolation		
PAMC No.	26583	26600
Scientific name of strain	*Cladonia pyxidata*	*Cetraria* sp.
Media for isolation	R2A agar	MY agar
Temperature	10 °C	10 °C
Culture duration	10 days	10 days
Colony morphology		
KOPRI No.	26583	26600
Media for experiment	MY	MY
Temperature	10 °C	10 °C
Culture duration	10 days	10 days
Form	Circular	Circular
Elevation	Convex	Convex
Margin	Entire	Entire
Surface	Glistening	Glistening
Optimum temperature (ºC)		
4	++	++
10	++	++
15	+	+
20	+	+
25	–	–
30	–	–
37	–	–
Days for temp. test	7 days	7 days
Media for temp. test	0.1× NA and MY	0.1× NA and MY
Optimal growth temp	4–10 °C	4–10 °C

### Genome assembly and phylogeny of two *Mrakia* isolates

To date, two *M. gelida* isolates have been sequenced—one using both PacBio Sequel and Illumina HiSeq technologies (GCA_024345305.1), and the other using Illumina HiSeq alone (GCA_017654745.1). The assembly statistics for GCA_024345305.1 revealed 25 contigs with a total assembly size of 34.9 Mb, whereas GCA_017654745.1 consisted of 506 contigs with a total genome size of 21.6 Mb. However, no genome sequence of *M. robertii* has been reported to date.

We sequenced the genomes of both strains via Oxford Nanopore technology and polished the assemblies with high-quality Illumina short reads. For PAMC 26583, 1,111,469 Nanopore reads comprising 3,923,634,917 bases were obtained. For PAMC 26600, 2,041,629 Nanopore reads comprising 2,106,756,018 bases were obtained via PromethION sequencing. In addition, a total of 7,229,390 Illumina short reads (1,091,637,890 bases) and 8,231,262 Illumina short reads (1,242,920,562 bases) were generated for PAMC 26583 and PAMC 26600, respectively (Supplementary Table S1). Before we performed the assembly, we estimated the genome size via GenomeScope2.0^16^ on the basis of Illumina short reads and predicted genome sizes of 34.45 Mb for PAMC 26583 and 29.14 Mb for PAMC 26600 (Supplementary Figure S2).

Nanopore reads for the two yeast strains were assembled via Nextdenovo^17^, and the assembled genome size of PAMC 26583 was 34.082 Mb, which is similar to both the previously reported GCA_024345305.1 assembly and the predicted genome size. The assembly contained 28 scaffolds, with 23 scaffolds longer than 100 kb, comprising a total scaffold length of 33.9 Mb. The assembled genome of PAMC 26600, identified as *M. robertii*, was 28.729 Mb in size, with 13 of the 15 scaffolds exceeding 100 kb, representing approximately 98% of the total scaffold length. These assembled genome sizes are also consistent with the predicted genome size (Table 2). To validate the genome assemblies, we performed BUSCO analysis via the Basidiomycota database^18^. The BUSCO completeness scores for PAMC 26583 and PAMC 26600 were 91.4% and 92.1%, respectively, with 8.1% and 7.5% missing BUSCOs, respectively (Table 2). The *de novo* repeat sequences were searched via RepeatModeler^19^, and the repeat sequences comprised 10.3% and 7.57% of the genome sequences of PAMC 26583 and PAMC 26600, respectively (Table 2 and Supplementary Tables S2 and 3).

**Table 2:** Genome assembly and gene annotation.

		PAMC 26583	PAMC 26600
Assembly statistics	number of contig	28	15
	contig L50 (Mbases)	2.043	2.493
	Max contig length (Mbases)	3.222	4.983
	Total contig length (Mbases)	34.211	28.976
	GC content (%)	55.8	55.4
Assembly validation	Complete BUSCOs (duplicated BUSCOs)	91.4 (0.7)	92.1 (0.8)
BUSCO completeness against Basidiomycota odb10	Fragmented BUSCOs	0.5	0.4
	Missing BUSCOs	8.1	7.5
Gene prediction	BRAKER3	8918	8131
Functional annotation	blastP	6897	6561
	eggNOG	6748	6410
	KEGG	3745	3702
	GO	3756	3288
	Pfam	6230	5914
Gene annotation Validation	Complete BUSCOs (duplicated BUSCOs)	90 (0.9)	91.5 (0.9)
BUSCO completeness against Fungi odb10	Fragmented BUSCOs	1.5	1.5
	Missing BUSCOs	8.5	7
*de novo* repeat sequence	Total repeat sequence (%)	10.3	7.57
tRNA	tRNAs decoding Standard 20 AA	130	119

NR, nonredundant protein database; Pfam, protein family; KEGG, Kyoto Encyclopedia of Genes and Genomes; GO, Gene Ontology; eggNOG, evolutionary genealogy of genes: Nonsupervised Orthologous Groups; BUSCO, benchmarking universal single-copy orthologs.

**Fig. 1:**
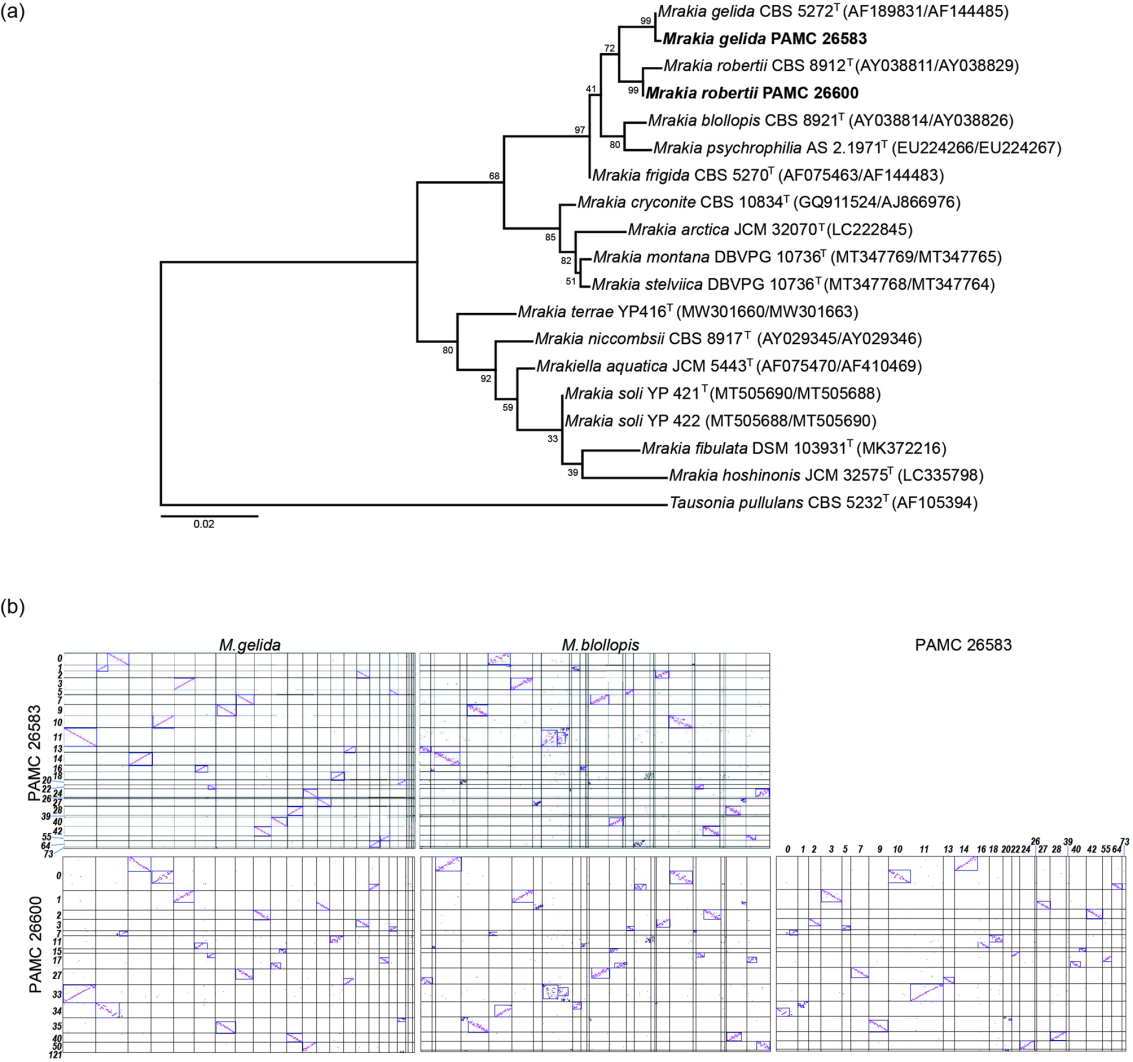
Phylogeny and synteny analysis of *Mrakia* genomes. (**a**) Phylogenetic tree based on the ITS region and D1/D2 domain sequences. This represents a maximum-likelihood analysis of the ITS region and D1/D2 domain sequences of two isolated strains and closely related species. The *Mrakia* strains investigated in this study are highlighted in bold. *Tausonia pullulans* CBS 5232^T^ was designated as the outgroup. The tree was reconstructed via maximum-likelihood analysis with MEGA7. In parentheses, the first number denotes the GenBank accession number for D1/D2, and the second number denotes the GenBank accession number for the ITS regions. (**b**) Syntenic relationships among the genomes of PAMC 26583, PAMC 26600, *Mrakia gelida* (GCA_024345305.1), and *Mrakia blollopis* (GCA_000950635.1) were analyzed via SyMAP. Each dot represents an anchor point indicating sequence similarity, whereas the blue boxes denote syntenic blocks identified by the SyMAP algorithm. The numbers along the axes correspond to the contig numbers of the assemblies for PAMC 26583 and PAMC 26600. The left and middle panels show synteny comparisons of *M. gelida* and *M. blollopis* with both PAMC 26583 and PAMC 26600, and shows more extensive synteny between PAMC 26583 and *M. gelida*. The right panel shows synteny between PAMC 26583 and PAMC 26600. The results highlight conserved and rearranged regions among cold-adapted *Mrakia* species.

To determine the taxonomic position of the two Arctic yeast strains PAMC 26583 and PAMC 26600 more precisely, phylogenetic analysis was performed via the internal transcribed spacer (ITS) region and the D1/D2 domain sequences of the large subunit rRNA gene from the assembled genome sequences. The maximum-likelihood tree included the two isolates and closely related species within the genus *Mrakia*. *Tausonia pullulans* CBS 5232ᵀ was designated as the outgroup. The phylogenetic tree (Fig. 1a) revealed that strain PAMC 26583 clustered with *Mrakia gelida* CBS 5272, whereas strain PAMC 26600 clustered with *Mrakia robertii* CBS 8912, each with high bootstrap support values (99% and 99%, respectively). These results confirmed the identification of PAMC 26583 as *M. gelida* and PAMC 26600 as *M. robertii*.

### Genomic synteny with other yeast genomes

PAMC 26583 showed high similarity to *M. gelida* based on ITS sequence comparison. When the genomic contigs of PAMC 26583 were compared with those of *M. gelida* (GCA_024345305.1), dot plot analysis revealed 22 syntenic blocks (Fig. 1b). The clustered hits covered 85.7% of the *M. gelida* genome and 85.6% of the PAMC 26583 genome, indicating high genomic similarity between the two strains. In contrast, compared with those of *M. blollopis* (GCA_000950635.1), both the size of the syntenic blocks and the coverage of the aligned regions were reduced. The number of cluster hits increased, but the size of the clusters and the proportion of the genome they covered decreased.

PAMC 26600 presented relatively low similarity to the available yeast genomes. Compared with *M. gelida* (GCA_024345305.1), the cluster hits covered 63.5% of the *M. gelida* genome and 74.6% of the PAMC 26600 genome. In the comparison between PAMC 26583 and PAMC 26600, the cluster hits covered 64.5% of the PAMC 26583 genome and 75.0% of the PAMC 26600 genome. These results suggest that the genome of PAMC 26600 is distinct from previously reported *Mrakia* genomes and will serve as a valuable reference for fungal genomics.

### Organellar genome assembly and annotation

To obtain the mitochondrial genome, we first reassembled the Nanopore reads into contigs via the Flye assembler^20^ and selected the candidate mitochondrial contigs with read coverage and tblastn search results against the NCBI nr database. One candidate contig for the mitochondrial genome was initially obtained for the PAMC 26583 strain, and two candidate contigs were obtained for PAMC 26600. To refine the mitochondrial reads, we mapped the Nanopore reads to the candidate contigs and then randomly subsampled the Nanopore reads to approximately 100× coverage of the mitochondrial genome. The subsampled reads were subsequently assembled into a single linear contig for each strain, PAMC 26583 and PAMC 26600. The resulting mitochondrial genomes were validated by remapping the randomly selected Nanopore reads to the assembled mitochondrial genomes using minimap2^21^ and visualizing the mapping results via IGV^22^ (Supplementary Figure S3). The linear structure of the mitochondrial genomes was inferred from the absence of overlapping terminal repeats and further confirmed by continuous coverage at contig ends in the Nanopore read mapping results (Supplementary Fig. S3).

**Fig. 2:**
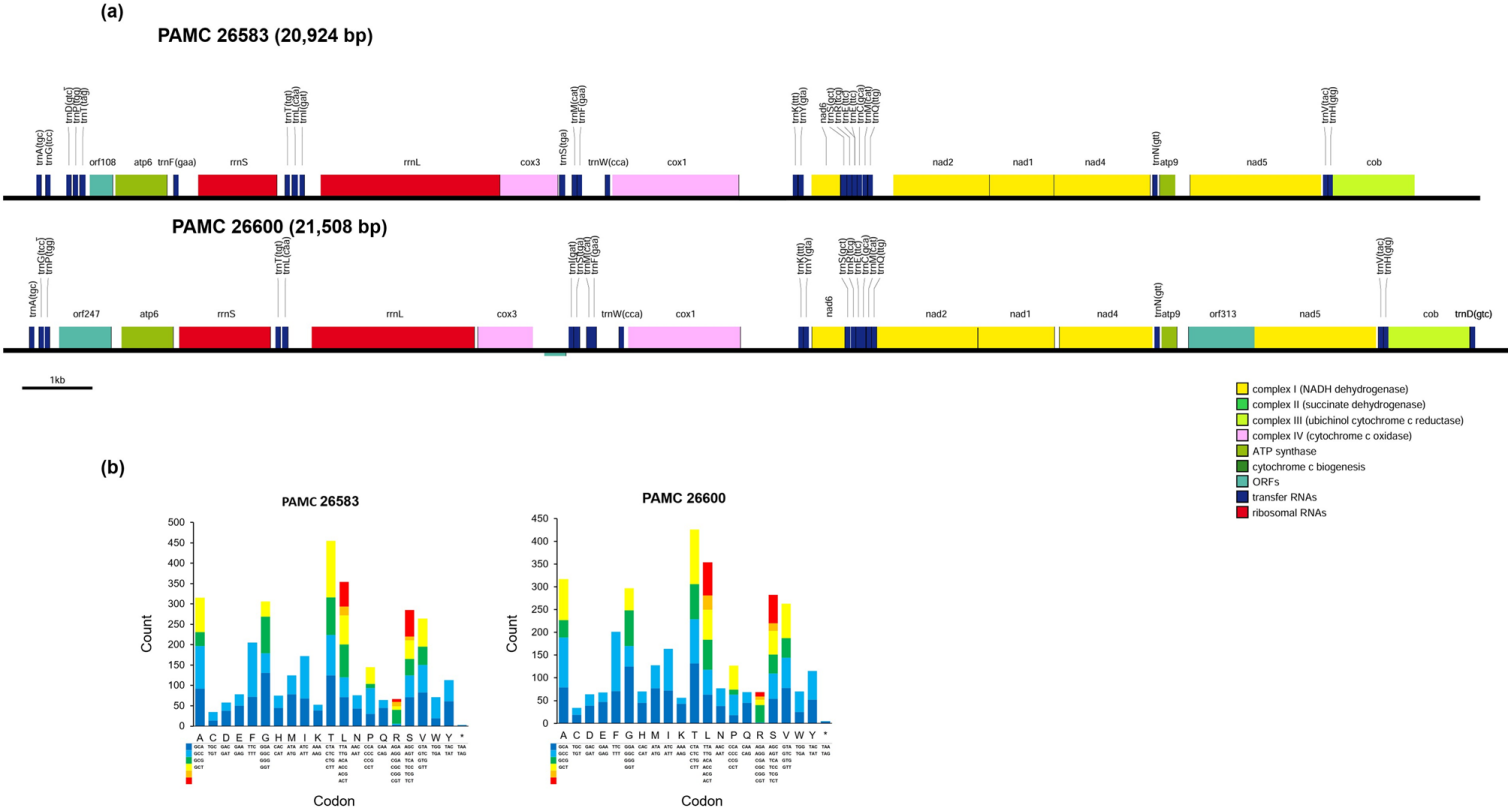
Mitogenomes of the two yeast strains. (**a**) The mitogenome is 20,924 bp and 21,508 bp long and contains 12 and 13 protein-coding genes, two rRNA genes and 24 and 22 tRNA genes. The types of genes are represented by boxes of different colors (legend in captions). The mitogenomes of these two strains are linear in terms of their assembly. (**b**) Codon usage in the mitogenomes of two *Mrakia* species. The frequency of codon usage is plotted on the y-axis.

The linear maps of the mitochondrial genome of PAMC 26583, generated via OGDRAW^23^, revealed that the genome size of PAMC 26583 was 20,924 bp, with a GC content of 45.0%, whereas that of PAMC 26600 was 21,508 bp, with a GC content of 46.3% (Fig. 2). In PAMC 26583, the mitochondrial genome contains two *cox* genes (*cox1* and *cox6*), four *nad* genes (*nad1*–*nad2*, *nad4*, and *nad6*), two *atp* genes (*atp6* and *atp9*), two ribosomal RNA genes (*rns* and *rnl*), and a *cob*. In addition, 24 tRNA genes corresponding to 20 amino acids were identified, along with 2 additional open reading frames (ORFs).

In PAMC 26600, 22 tRNA genes encoding 20 amino acids were identified, along with an additional ORF. The remaining genes were identical to those found in PAMC 26583.

### Gene annotation of the two *Mrakia* strains

We performed gene annotation via BRAKER3^24^ with both RNA-Seq and protein reference data. The RNA-Seq data were mapped to the genome to define expressed gene regions, and protein sequences from ten *basidiomycete* fungal species from the fungal genomics resource of JGI (*Cryptococcus curvatus* ATCC 20509, *Cryptococcus neoformans* var neoformans JEC21, *Dioszegia hungarica* PDD-24b-2, *Fellomyces penicillatus* Phaff54-35, *Filobasidium floriforme* CBS 6241, *Kockovaella imperatae* NRRL Y-17943, *Naganishia vishniacii* ANT03-052, *Pseudozyma antarctica*, *Ustilago maydis* 521, and *Phaffia rhodozyma* UBV-AX4 were used as reference proteins. A total of 8,919 and 8,131 genes were predicted by BRAKER3 for PAMC 26583 and PAMC 26600, respectively (Table 2).

Functional annotation was conducted via BLASTP and EggNOG-mapper^25^ against the NCBI nr database and the EggNOG database^26^. Functional assignments were obtained for more than 75% of the predicted genes via EggNOG and BLASTP, and Gene Ontology (GO) terms were assigned for approximately 40% of the genes based on EggNOG annotations.

BUSCO analysis was performed to assess the completeness of the annotated gene sets via the Fungi dataset. The gene set completeness values for PAMC 26583 and PAMC 26600 were 90.0% and 91.5%, respectively, with 8.5% and 7.0% missing BUSCOs, respectively. The gene set completeness was consistent with the genome completeness values obtained from the initial BUSCO analysis (Table 2).

### Phylogenetic analysis of the tremellomycetes class, including PAMC 26583 and PAMC 26600

**Fig. 3:**
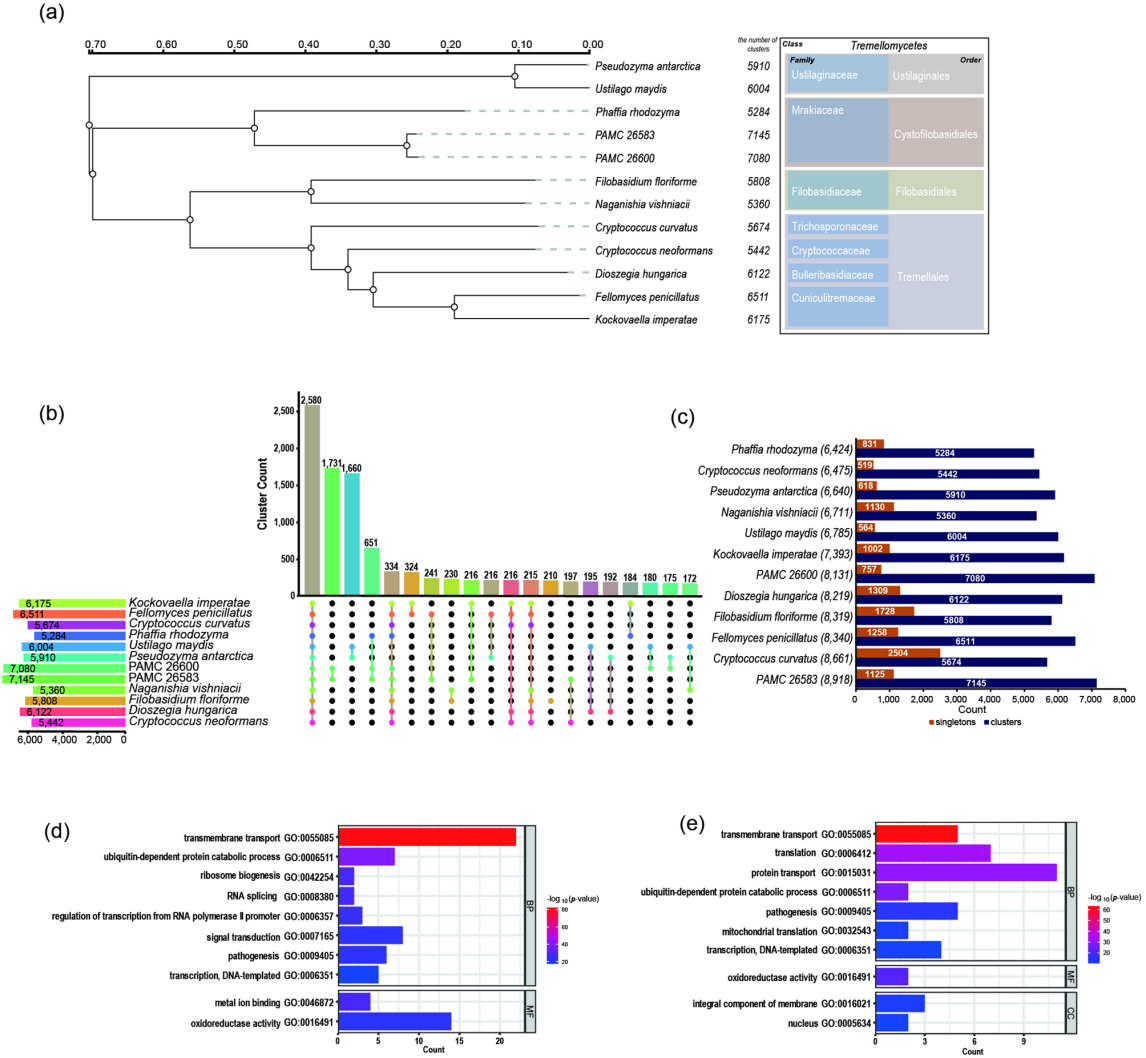
Phylogenetic tree and orthologous gene cluster analysis of 12 fungal genomes. (**a**) FastTree2 was used to construct a phylogenetic tree via the maximum likelihood method with the JTT + CAT evolutionary model. Protein sequences from 2,580 orthologous gene clusters were used for analysis. Both PAMC 26583 and PAMC 26600 are located within the family Mrakiaceae of the order Cystofilobasidiales, together with *P. rhodyzyma*. The number of orthologous groups assigned to each species is indicated next to each taxon. (**b**) UpSet plot showing the top 20 orthologous cluster intersections across species with the highest shared cluster counts. Each bar represents the number of orthologous clusters shared by the species indicated below with connected dots. The tallest bar (2,580 clusters) represents core orthologs shared among all 12 species. (**c**) Bar graph showing the number of orthologous clusters (blue) and singletons (orange) identified in each species. The total number of proteins is indicated in parentheses after each species name. PAMC 26583 and PAMC 26600 exhibited 7,145 and 7,080 orthologous clusters, respectively, with relatively low singleton counts (1,125 and 757), indicating a high proportion of conserved genes. This analysis highlights the distribution of shared and unique gene families across Tremellomycetes and related taxa. (**d**–**e**) Bar plots displaying significantly enriched Gene Ontology (GO) terms among orthologous gene clusters shared by (**d**) *Mrakia* species (*M. gelida* PAMC 26583 and *M. robertii* PAMC 26600; the second largest set of orthologous gene clusters) and by (**e**) species belonging to the order Cystofilobasidiales (4th largest set of orthologous gene clusters). Each bar represents a GO term, with its length indicating the number of assigned genes and color scale representing statistical significance (–log₁₀ p value). BP, MF, and CC denote Biological Process, Molecular Function, and Cellular Component, respectively.

A phylogenetic tree was constructed via FastTree2^27^ based on orthologous gene clusters, employing the maximum likelihood method with the JTT + CAT evolutionary model. As shown in Fig. 3, both PAMC 26583 and PAMC 26600 with *Phaffia rhodozyma* were placed within the family Mrakiaceae of the order Cystofilobasidiales.

The species tree also shows broader relationships among the Tremellomycetes class. *Pseudozyma antarctica* and *U. maydis* 521 clustered together within the family Ustilaginaceae under the order Ustilaginales. Species such as *F. floriforme* CBS 6241 and *N. vishniacii* ANT03-052 grouped into the family Filobasidiaceae of the order Filobasidiales, whereas *C. curvatus* ATCC 20509, *C. neoformans* var. *neoformans* JEC21, and *D. hungarica* PDD-24b-2 formed a separate cluster within the order Tremellales. Additionally, *F. penicillatus* Phaff54-35 and *K. imperatae* NRRL Y-17943 were assigned to the family Cuniculitremaceae under the order Tremellales (Fig. 3a).

### Orthologous group analysis of twelve yeast strains

To identify orthologous gene groups in the two yeast strains via OrthoVenn3^28^, we selected 10 fungal genomes from the Fungal Genomics Resource of JGI that had gene annotations and fewer than 50 scaffolds. Both yeast strains belong to the order Cystofilobasidiales within the class Tremellomycetes; therefore, we downloaded high-quality genome data from species closely related to this order. Genome data for *P. antarctica* and *U. maydis* 521 (order Ustilaginales), *F. floriforme* CBS 6241 and *N. vishniacii* ANT03-052 (order Filobasidiales), *C. curvatus* ATCC 20509, *C. neoformans* var neoformans JEC21, *D. hungarica* PDD-24b-2, *F. penicillatus* Phaff54-35, and *K. imperatae* NRRL Y-17943 (order Tremellales) were used for this analysis. Genome data for *P. rhodozyma* UBV-AX4 (order Cystofilobasidiales) were also included (Supplementary Table S4).

The protein sequences used in the BRAKER3 annotation pipeline for the two yeast strains were employed for this orthologous analysis. Prior to analysis, the transcript variants were filtered such that only representative proteins were retained. Orthologous analysis was performed via OrthoVenn3 with the OrthoMCL algorithm.

A total of 2,580 orthologous gene clusters were identified, which included proteins from all 12 species (Fig. 3b). For PAMC 26583, proteins were distributed across 7,145 clusters, with 1,125 singletons. For PAMC 26600, proteins were distributed across 7,080 clusters, with 757 singletons (Fig. 3c). The orthologous gene cluster shared by PAMC 26583 and PAMC 26600 represented the second largest cluster, comprising 1731 clusters. The enriched GO terms of this cluster included transmembrane transport (GO:0055085), ubiquitin-dependent protein catabolic process (GO:0006511), metal ion binding (GO:0046872), ribosome biogenesis (GO:0042254), and RNA splicing (GO:0008380) (Fig. 3d). The orthologous gene cluster shared by *U. maydis* 521 and *P. antarctica* represented the third largest cluster, whereas the cluster containing proteins from species of the order Cystofilobasidiales represented the fourth largest cluster. These clusters were enriched for GO terms such as transmembrane transport (GO:0055085), translation (GO:0006412), protein transport (GO:0015031), and ubiquitin-dependent protein catabolic process (GO:0006511) (Fig. 3e).

### Gene contraction and expansion analysis among twelve yeast strains

To investigate gene family expansion and contraction during evolution, we used CAFE5^29^ to estimate changes in gene family size based on gene family size and divergence time. The divergence times for two pairs of species were obtained from TimeTree5^30^: the divergence time between Cystofilobasidiales and Ustilaginales was estimated at 442 million years (Myr), and that between Cystofilobasidiales and Tremellales was estimated at 301 Myr.

**Fig. 4:**
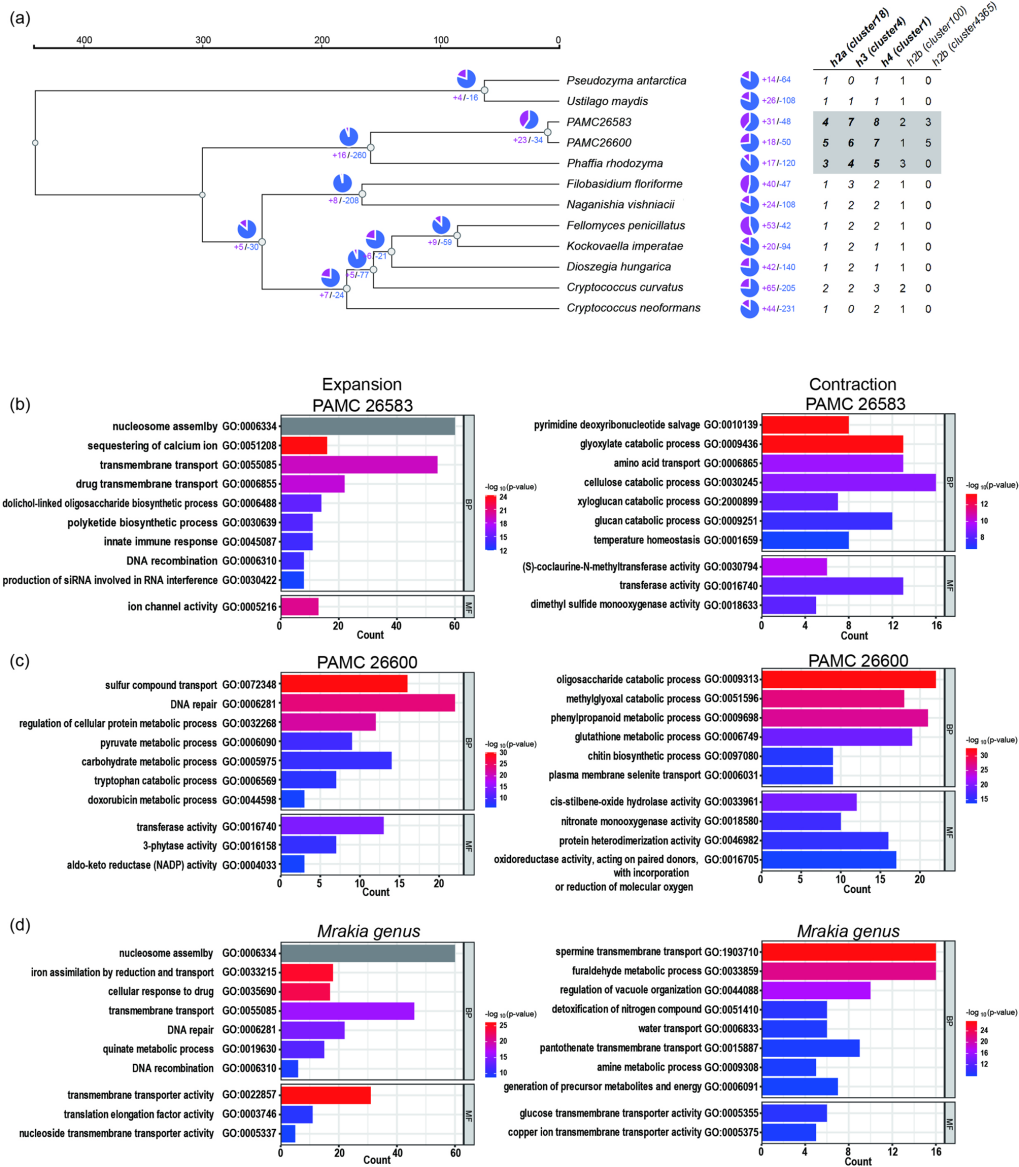
Lineage-specific gene family expansion and contraction across twelve yeast genomes and GO enrichment analysis of *Mrakia* strains and related lineages. (**a**) Gene family gains (purple) and losses (blue) are indicated at each branch of the phylogenetic tree. The numbers represent the total number of expanded (+) or contracted (−) gene families along each lineage, as inferred by CAFE5. The phylogenetic tree was constructed via FastTree (v2.1.10) on the basis of aligned orthologous gene families, and species divergence times were inferred via TimeTree v5. The number of histone genes is shown in the right panel; bold text denotes expanded clusters, and gray shading indicates the total number of histone genes in the Cystofilobasidiales lineage. (b-d) GO enrichment analysis of expanded and contracted gene families in *Mrakia* strains and related lineages. Bar plots display significantly enriched Gene Ontology (GO) terms among expanded (left) and contracted (right) gene families identified by CAFE5 in PAMC 26583 (**b**), PAMC 26600 (**c**), and their ancestral lineages (**d**). Each bar represents a GO term, with its length indicating the number of genes assigned and color scale representing statistical significance (–log₁₀ p value). The functional categories enriched in the expanded families included transmembrane transport, DNA repair, sulfur compound transport, and iron assimilation, suggesting enhanced environmental adaptability. In contrast, contracted gene families were enriched in pathways such as oligosaccharide catabolism, methylglyoxal metabolism, and phenylpropanoid degradation, indicating possible metabolic specialization in cold environments. BP, MF, and CC denote biological process, molecular function, and cellular component.

In the PAMC 26583 lineage, 31 gene families were expanded, with enriched GO terms, including nucleosome assembly (GO:0006334, BP), sequestration of calcium ions (GO:0051208, BP), and iron channel activity (GO:0005216, MF) (Fig. 4a and b). A total of 48 gene families were contracted, and the enriched GO terms included pyrimidine deoxyribonucleotide salvage (GO:0010139, BP), glyoxylate catabolic process (GO:0009436, BP), and (S)-coclaurine-N-methyltransferase activity (GO:0030794, MF).

In the PAMC 26600 lineage, 18 gene families were expanded, with enriched GO terms, including sulfur compound transport (GO:0072348, BP), DNA repair (GO:0006281, BP), and regulation of cellular protein metabolic process (GO:0032268, BP) (Fig. 4a and c). Moreover, 50 gene families were contracted, and the enriched GO terms included oligosaccharide catabolic process (GO:0009313, BP), methylglyoxal catabolic process (GO:0051596, BP), and phenylpropanoid metabolic process (GO:0009698, BP).

In the *Mrakia* genus lineage, 23 gene families expanded, and 34 gene families contracted (Fig. 4a). The expanded families were enriched for nucleosome assembly (GO:0006334, BP), transmembrane transporter activity (GO:0022857, MF), and iron assimilation by reduction and transport (GO:0033215, BP) (Fig. 4a and d). The contracted gene families were enriched for GO terms such as spermine transmembrane transport (GO:1903710, BP), furaldehyde metabolic process (GO:0033859, BP), and regulation of vacuole organization (GO:0044088, BP).

The GO term nucleosome assembly (GO:0006334, BP) was enriched from the Cystofilobasidiales lineage to the PAMC 26583 lineage. This enrichment is associated with the expansion of clusters 1, 4, and 18, which were annotated as histones H4, H3, and H2A, respectively. Although histone H2B was not identified within expanded gene clusters in the CAFE analysis, its copy number was greater than that of the other histones, and some H2B genes were found exclusively in the *Mrakia* genus lineage (Fig. 4a).

### Carbohydrate-active enzymes and peptidases

**Fig. 5:**
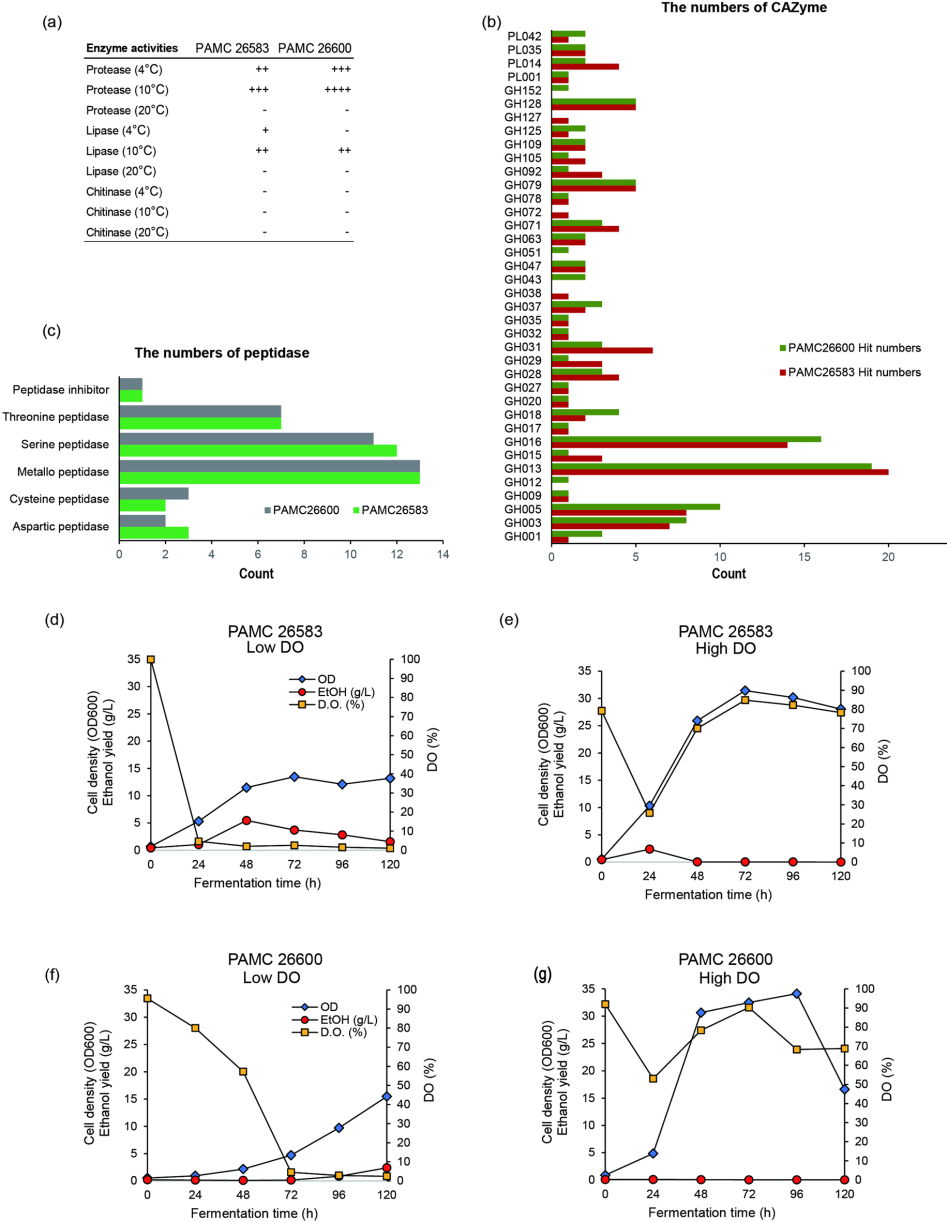
Enzymatic activities and distribution of CAZymes and peptidases in PAMC 26583 and PAMC 26600. (**a**) Enzymatic activity assays for protease, lipase, and chitinase at 4, 10, and 20 °C. The strains were cultivated on 0.1× nutrient agar (NA) plates, and their activities were assessed after 7 days of incubation. Activities are indicated by plus signs (+), with more plus signs denoting higher activity; “-” indicates no detectable activity. (**b**) Numbers of carbohydrate-active enzymes (CAZymes) identified in each strain via dbCAN2. The functional categories are labeled with CAZy family names (PL, polysaccharide lyase; GH, glycoside hydrolase), and the bar colors indicate strain-specific hit numbers (green, PAMC 26600; red, PAMC 26583). (**c**) Numbers of peptidases in each strain identified via MEROPS, grouped by functional class. The green bars indicate PAMC 26583, and the gray bars indicate PAMC 26600. (**d**‒**g**) Ethanol production, cell density, and dissolved oxygen levels during fermentation by the *Mrakia* strains PAMC 26583 and PAMC 26600 under different dissolved oxygen (DO) conditions. Fermentation profiles were monitored for 120 h under low-DO (< 5%) and high-DO (> 50%) conditions. Plots show changes in cell density (OD_600_, blue diamonds), ethanol concentration (g/L, red circles), and dissolved oxygen level (%, yellow squares) for PAMC 26583 under (**d**) low-DO and (**e**) high-DO conditions and for PAMC 26600 under (**f**) low-DO and (**g**) high-DO conditions. PAMC 26583 showed significantly greater ethanol production under low DO, reaching a maximum of 5.46 g/L at 48 h, whereas both strains exhibited negligible ethanol production under high DO despite comparable or greater cell growth.

Since both strains can grow at subzero temperatures but cannot grow above 20 °C, their cold-active enzymes may have potential applications for industrial use. The enzymatic activities of protease, lipase, and chitinase were analyzed at 4, 10, and 20 °C (Fig. 5a). Protease and lipase activities were detected only at temperatures below 10 °C in both strains, whereas chitinase activity was not detected at any temperature. We also analyzed the protein sequences via dbCAN2 and MEROPS to identify candidate CAZymes and peptidases (Fig. 5b and c). Using dbCAN2, 114 CAZymes belonging to 34 functional categories were identified in PAMC 26583, and 113 CAZymes in 35 categories were identified in PAMC 26600 (Supplementary Table S5). In both strains, glycoside hydrolases (GHs) constituted the most significant proportion of CAZymes, followed by polysaccharide lyases (PLs). Using MEROPS, we identified 38 peptidases in PAMC 26583 and 27 in PAMC 26600 (Supplementary Table S6), with metallopeptidases being the most abundant category.

### Alcohol production

We evaluated the ethanol production capabilities of PAMC 26583 and PAMC 26600 under controlled dissolved oxygen (DO) conditions at 12 °C (Fig. 5d–g).

Under low DO conditions (< 5%), PAMC 26583 demonstrated significant ethanol production. The ethanol concentration increased rapidly during the initial 48 h, reaching 5.46% (v/v), with the corresponding cell growth reaching a cell density (OD_600_) = 11.5. After prolonged incubation (120 h), the ethanol concentration decreased to 2.4% (v/v), possibly due to ethanol consumption or metabolic shifts, while the cell density remained stable at OD_600_ = 13.17. The dissolved oxygen levels continuously decreased during fermentation, dropping from 4.69% at 24 h to 1.04% at 120 h.

In contrast, under high DO conditions (> 50%), ethanol production by PAMC 26583 was minimal. The maximum ethanol concentration observed was only 2.38 g/L at 24 h, and ethanol was almost undetectable after 96 h. Although cell growth reached higher OD_600_ values (up to 31.4 at 74 h), ethanol synthesis was strongly suppressed under these aerobic conditions, suggesting that oxygen availability plays a critical role in regulating fermentation pathways in this strain.

For PAMC 26600, a similar oxygen-dependent ethanol production pattern was observed, although the overall ethanol yields were lower than those of PAMC 26583. Under low-DO conditions, the ethanol concentration reached 2.4 g/L (approximately 0.3% v/v) after 120 h, while the cell density reached OD_600_ = 15.47. Under high DO conditions, ethanol production was negligible throughout the experiment, with maximum ethanol concentrations remaining below 0.07 g/L.

These results indicate that both strains, particularly PAMC 26583, possess the capacity for low-temperature ethanol fermentation under limited oxygen availability. The superior ethanol production capacity of PAMC 26583 under hypoxic conditions suggests its potential as a cold-active yeast for industrial applications such as lager beer production or other low-temperature fermentations.

## Discussion

Cold-adapted yeasts represent an important group of extremophilic fungi with significant ecological roles and potential industrial applications because of their ability to function at low temperatures. In this study, we isolated and sequenced two cold-adapted *Mrakia* strains, PAMC 26583 and PAMC 26600, from Arctic lichens. PAMC 26583 was identified as *Mrakia gelida*, and PAMC 26600 was identified as *Mrakia robertii* based on the ITS region and D1/D2 domain sequences of the large-subunit rRNA. While genomic data for *M. gelida* have been previously reported, this study presents, to our knowledge, the first high-quality genome assembly of *M. robertii*.

Both *Mrakia* strain genomes were assembled via Nanopore long-read sequencing combined with Illumina short-read polishing, resulting in highly contiguous and nearly complete assemblies. The genome completeness of BUSCO analysis was greater than 90%, and genome size estimation via GenomeScope 2.0 was consistent with the assembly results, supporting the accuracy of the assemblies. Notably, we successfully assembled and annotated linear mitochondrial genomes for both strains. PAMC 26583 and PAMC 26600 belong to the phylum Basidiomycota, in which 693 mitochondrial genomes have been reported, ranging in size from 11,198 bp to 272,497 bp, demonstrating substantial variation^31^. Within the class Tremellomycetes, 65 mitochondrial genomes are currently available in the NCBI database; however, only a single unannotated mitogenome of *Tausonia pullulans* (OX637640.1) was previously reported for the order Cystofilobasidiales^32^. This study therefore provides the first annotated mitochondrial genomes and the second overall genome sequences for this order.

The fungal mitochondrial genome typically encodes 14 highly conserved core genes^33,34^, including NADH dehydrogenase subunits (*nad1*–*nad6* and *nad4L*) for Complex I, the cytochrome b gene (*cob*) for Complex III, three cytochrome c oxidase subunit genes (*cox1*, *cox2*, and *cox3*) for Complex IV, and ATP synthase subunits (*atp6*, *atp8*, and *atp9*). It also contains genes for small and large ribosomal RNA subunits (*rns* and *rnl*)^34^. In this study, the mitochondrial genomes of PAMC 26583 and PAMC 26600 were predicted to be linear, containing 36 and 35 genes, respectively. However, *nad3*, *nad4L*, *cox2*, and *atp8* were absent from their genomes. Similarly, in *T. pullulans*, *cox2* and *atp8* were also not identified, suggesting that these absences may be characteristic of the mitochondrial genomes in the Mrakiaceae family. Analysis of the *nad2* gene sequences of both *Mrakia* strains suggested that *nad3* may be fused with *nad2* (Supplementary Fig. S4). Given that *nad2* and *nad3* are not known to be expressed as fusion proteins, additional experimental validation will be necessary to confirm this observation.

Compared with those of other Basidiomycota, the mitochondrial genomes of PAMC 26583 and PAMC 26600 are relatively small, with intergenic regions comprising less than 30% of the genome (26.9% and 29.7%, respectively)^31^. Although the mitochondrial genome of *T. pullulans* contains more genes, including *nad3* and *nad4L*, than either *Mrakia* strain does (Supplementary Fig. S5), its overall size (18,821 bp) is smaller, with even shorter intergenic regions (23.6%). *T. pullulans* is also psychrotolerant, and previous studies have suggested associations between mitochondrial function and cold adaptation^35^, as well as between mitogenome size and adaptation to low temperatures^36^. Therefore, further investigation of the mitochondrial genomes of these two cold-adapted yeasts may provide valuable insights into the evolutionary and physiological mechanisms underlying their psychrophily.

Comparative genomic analyses revealed strong synteny between the PAMC 26583 and previously reported *M. gelida* genomes, whereas PAMC 26600 presented lower synteny with other *Mrakia* species, indicating evolutionary divergence within Cystofilobasidiaceae. Orthologous gene clustering with related Tremellomycetes species revealed that PAMC 26583 and PAMC 26600 form a distinct clade within Cystofilobasidiaceae, supporting their phylogenetic placement.

After functional gene annotation, we examined gene family expansion and contraction within the Cystofilobasidiaceae lineage. We identified enriched GO terms such as nucleosome assembly, DNA repair, metal ion transport, and transmembrane transporter activity in the expanded gene families. The enriched GO terms, such as nucleosome assembly (GO:0006334, BP) and DNA repair (GO:0006281, BP), were largely attributable to the expansion of histone genes. The number of histone genes in the Cystofilobasidiales lineage was considerably greater than that in other fungi, and additional histone H2B genes were also identified within the *Mrakia* lineage. Although the effects of these distinct patterns and increased histone gene copy numbers on organismal evolution remain unclear, functional studies using histone gene mutants in these strains may help to elucidate the roles of histone gene expansion in adaptation and physiology.

*Mrakia* strains, including *M. robertii* and *M. gelida*, are known to produce high levels of polyunsaturated fatty acids (PUFAs), comprising approximately 65% of total lipids. In particular, strains grown at − 1 °C have been reported to contain up to 49% linolenic acid^7^. As PUFA synthesis typically involves Δ9, Δ12, and Δ15 fatty acid desaturases, we examined orthologous gene clusters and identified *Mrakia*-specific Δ9 fatty acid desaturase (cluster 8579) and Δ12 fatty acid desaturases (clusters 8929 and 7692) (Supplementary Table 7). Although the biochemical activities of these enzymes remain to be verified, these *Mrakia*-specific orthologous groups likely contribute to PUFA biosynthesis and cold adaptation. Previous genomic studies of the cold-adapted yeast *Mrakia psychrophila* have reported a large number of genes encoding Major Facilitator Superfamily (MFS) transporters (133)^37^, which facilitate nutrient uptake and stress adaptation in cold environments. Similarly, in *M. gelida* PAMC 26583 and *M. robertii* PAMC 26600, we identified 58 and 59 MFS transporter genes, respectively—higher than those of most related yeast species (16–44)—suggesting that MFS-mediated membrane transport may play an important role in environmental adaptation under cold and nutrient-limited conditions.

In contrast to other Antarctic yeasts such as *Naganishia vishniacii*, *Phenoliferia glacialis*, and *Glaciozyma antarctica*, which harbor antifreeze glycoprotein (AFGP) or antifreeze protein (AFP) genes^38^, no homologs of AFGP, AFP, or ice-binding proteins (IBPs) were detected in either *Mrakia* genome. Additional HMM and protein similarity searches confirmed the absence of antifreeze-related domains, suggesting that *Mrakia* may rely on alternative mechanisms for freezing tolerance. Antioxidant defense-related genes, including *Mrakia*-specific clusters 6841 and 7507 encoding superoxide dismutase, were also identified, although *Mrakia*-specific catalase and glutathione peroxidase genes were not found. Furthermore, both *Mrakia* genomes encode two trehalose synthase genes, which may contribute to osmoprotection and cryoprotection under cold stress, but *Mrakia*-specific trehalose synthase was not identified.

*Mrakia* species are known to secrete cold-active enzymes such as lipases, proteases, and cellulases that remain functional across a broad temperature range (− 3 °C to 20 °C) ^39^. These cold-active enzymes show considerable potential for industrial applications. The lipase activity of *M. blollopis* SK-4 has been reported to play a pivotal role in removing milk fat curdles from sewage under low-temperature bioremediation conditions^10^. Similarly, cold-active proteases are already used in low-temperature detergents for industrial purposes^40^. In the food industry, particularly during cheese and dairy maturation, low-temperature proteolysis contributes to flavor development while preserving volatile aroma compounds—processes for which cold-active proteases are especially desirable^40^. *Mrakia* species, which are not on the GRAS (Generally Recognized As Safe) list of organisms, may therefore represent promising yeast-based sources of such enzymes. In the present study, we examined lipase, protease, and chitinase activities at 4–20 °C and confirmed that both *M. gelida* PAMC 26583 and *M. robertii* PAMC 26600 exhibited measurable lipase and protease activities at 4–10 °C, supporting their potential utility in cold-temperature industrial processes.

Furthermore, owing to their high polyunsaturated fatty acid (PUFA) biosynthetic capacity, *M. robertii* and related *Mrakia* species produce lipids with fatty-acid chain lengths and unsaturation levels well suited for biodiesel conversion, yielding fuels with excellent cold-flow properties^41^. As *Mrakia* species are psychrophilic, biomass and lipid production are particularly suitable for low-temperature environments, enhancing their potential for sustainable industrial use. In addition, both PAMC 26583 and PAMC 26600 demonstrated ethanol production under low-temperature fermentation conditions, highlighting their potential application in the low alcohol brewing and wine industries^42^. Candidate genes encoding alcohol dehydrogenases were identified in both strains—*g489.t1* and *g5238.t1* in PAMC 26600, and *g857.t1* and *g3287.t1* in PAMC 26583. Collectively, these findings provide new insights into the genomic characteristics, metabolic capacities, and evolutionary adaptations of *Mrakia* species in cold environments, emphasizing their promise as versatile biotechnological resources.

## Conclusion

This study provides high-quality genomic resources for two cold-adapted *Mrakia* strains, including the first genome of *M. robertii* and the first reported mitochondrial genome sequence for the genus *Mrakia*. Through comprehensive comparative genomics, phylogenetic analysis, and gene family evolution analysis, we characterized the unique genetic features of these strains. Our findings highlight their metabolic versatility, potential industrial applications in cold-active enzyme production and fermentation, and evolutionary divergence within Mrakiaceae. The newly generated genomic data will serve as valuable references for future studies on the adaptation mechanisms of psychrophilic yeasts and for the development of biotechnological applications. The mitochondrial genomes of PAMC 26583 and PAMC 26600 are the only ones reported in the genus *Mrakia*, representing an essential taxonomic resource that fills a gap in the class Tremellomycetes.

## Materials and methods

### Yeast strains and culture conditions

The *Mrakia* strains PAMC 26583 and PAMC 26600 were isolated from Arctic lichen samples. The lichen samples were suspended in 10 mL of sterile saline and diluted 1:10; 100 µL of each dilution from each lichen suspension was spread onto malt-yeast (MY) agar and Reasoner’s 2 A (R2A) agar plates and maintained at 10 °C for 10 days. As no significant difference in growth was observed among various media (R2A, 0.1 × R2A, MY, NA, 0.1 × NA) at 15 °C for 7 days, two *Mrakia* strains were grown on MY agar plates at 10 °C for 14 days for colony morphology observation. To identify the optimum temperature, two *Mrakia* strains were grown on 0.1 × nutrient agar (NA) and MY agar plates at 4, 10, 15, 20, 25, or 30 °C for 7 days. Enzyme activities (protease, lipase, and chitinase) were tested at 4 °C and 10 °C in 0.1× NA media for 7 days.

### DNA extraction and sequencing

Genomic DNA was extracted via a DNeasy Mini Kit (Qiagen), followed by quality and quantity assessment via Nanodrop, Qubit, and gel electrophoresis. For long-read sequencing, libraries were prepared via an ONT 1D ligation sequencing kit (SQK-LSK109) following the manufacturer’s protocol. Sequencing was performed on an Oxford Nanopore PromethION platform using FLO-PRO002 flow cells (R9.4.1 chemistry). Base-calling was conducted with Guppy (v5.0.16), and the reads were filtered and trimmed via Porechop (v0.2.4). Illumina paired-end reads (2 × 150 bp) were obtained via the HiSeq platform for polishing and genome size estimation. All sequencing procedures were performed by Phyzen Co. Ltd. (Seongnam, Korea).

### Identification of yeasts

The isolated strains were initially identified on the basis of internal transcribed spacer (ITS) gene region sequences. The ITS gene region was amplified and sequenced using the primer pair ITS1/ITS4^43^ with Sanger sequencing technology. The sequencing results were assembled with the CAP contig assembler in BioEdit^44^ and searched via the Basic Local Alignment Search Tool (BLAST)^45^. For phylogenetic analysis, the ITS and D1/D2 region sequences of PAMC 26583 and PAMC 26600 were extracted from the draft genome sequence and concatenated. Those of closely related strains were downloaded as described by Park et al.. (2021)^5^. Sequence alignment was performed with MUSCLE^46^. Maximum likelihood with a G92 + G model was performed via MEGA7^47^. A bootstrap analysis with 1000 replicates was performed to estimate the confidence of the tree nodes. *Tausonia pullulans* CBS 5232T (AY029345/AY029316) was used as an outgroup in this analysis.

### Genome assembly and syntenic alignment

The Oxford Nanopore long reads were assembled via NextDenovo (v2.4.0)^17^ (https://github.com/Nextomics/NextDenovo) and polished via NextPolish (v1.3.1)^48^. The completeness of the assembled genome was evaluated by benchmarking universal single-copy orthologs (v5.1.3) (BUSCO)^18^. SyMAP (v4.0)^49^ was used to analyze the syntenic blocks among the fungal contigs [20], and dot plots were generated.

### Mitochondrial genome assembly

For mitochondrial genome assembly, nanopore reads were first assembled into contigs via Flye (v2.9.6)^29^, and candidate mitochondrial contigs were selected from the initial Flye assembly on the basis of coverage and tblastn results against the NCBI nr database. Nanopore reads were mapped to these contigs via minimap2 (v2.9)^21^ (“-a -x map-ont”), and 100× coverage reads were sampled via seqtk (r133) (“sample” option). The mitochondrial genomes were reassembled with Flye. The final assemblies were validated by read mapping nanopore reads with minimap2 and visualized with IGV tools (v2.16.1)^22^. Annotation was performed via MFannot (v2)^50^ and mitos (v2.1.9)^51^, and the resulting .sqn files were subsequently converted to GenBank format via Sequin (v16). These GenBank files were used for OGdraw (v1.3.1)^23^ to generate a mitochondrial map.

### Gene annotation

Gene prediction was performed via BRAKER3 (v3.0.8)^24^ in protein and transcript modes, integrating RNA-Seq reads and protein hints from ten Basidiomycetes (*C. curvatus*, *C. neoformans*, *D. hungarica*, *F. penicillatus*, *F. floriforme*, *K. imperatae*, *N. vishniacii*, *P. antarctica*, *U. maydis*, and *P. rhodozyma*). The functional annotations were derived via BLASTP^45^ against nr and EggNOG-mapper (v2.1.13)^25^ with EggNOG v5.0^26^ to assign gene functions and GO terms. The reference sequence included masked genome sequences obtained via RepeatMasker (v4.2.0)^52^ with a *de novo* repeat sequence. We verified the protein-coding gene annotation via BUSCO in protein mode against the Fungi_odb10 database. The functional annotation of the protein-coding genes was performed via EggNOG mapper (v.2.1.12) against the EggNOG database (v.5.0) and BLASTp (v.2.0.8) against the nr database.

### Repetitive sequence annotation

Repetitive sequences in the assembled genome were annotated via RepeatMasker (v4.0.7)^52^. A *de novo* repeat library for the identification of repeat elements was constructed via RepeatModeler (v1.0.11)^19^. The tRNA genes were identified via tRNAscan-SE (v2.0)^53^ with default parameter settings.

### Orthologous clustering, phylogeny, and gene family evolution

Protein sets from PAMC 26583, PAMC 26600, and ten Basidiomycetes, which were used in BRAKER3 as protein hints, were clustered via OrthoVenn3^28^ with OrthoMCL (e-value 1e-5, inflation 1.5). Species phylogeny was inferred with FastTree2^27^ via the JTT + CAT model from concatenated single-copy orthogroups. Gene family expansions and contractions were analyzed via CAFE5^29^, with divergence time calibration (442 Myr for Cystofilobasidiales–Ustilaginales; 301 Myr for Cystofilobasidiales–Tremellales) from TimeTree5^30^. Enriched GO terms among the changed gene families were identified within OrthoVenn3.

### CAZyme and peptidase identification

Carbohydrate-active enzymes (CAZymes) were identified via dbCAN2^54^ with default parameters.

The HMMER parser and HMM database (2019-09-05 release) were obtained from the dbCAN2 online repository (http://bcb.unl.edu/dbCAN2/download/).

For peptidase identification, we used the nonredundant MEROPS^55^ pepunit database, which contains all functional peptidase and inhibitor domains. Protein sequences were searched against the MEROPS pepunit database via DIAMOND (v2.1.13)^56^ BLASTP with the following parameters: “-k 1 -e 1e-10 --query-cover 80 --id 50” [47]. The MEROPS pepunit database was used to reduce nonspecific hits by focusing only on the functional units of peptidases and inhibitors.

### Ethanol fermentation experiments

To evaluate ethanol production, the *Mrakia* strains PAMC 26583 and PAMC 26600 were cultivated in YPD media under low (< 5%) and high (> 50%) dissolved oxygen (DO) conditions at 13 °C and 16 °C, respectively. Fermentation was carried out in a 7-L bioreactor (KF-7 L model, Kobiotech, Korea) with a working volume of 4 L, inoculated with 200 mL of seed culture. The seed cultures were grown at 15 °C for approximately 16 h, reaching an optical density (OD₆₀₀) of 0.85–2.50.

DO levels were regulated via a sealed fermentation system equipped with dissolved oxygen sensors and appropriate gas flow control or agitation speed. The cell density (OD₆₀₀), ethanol concentration (g/L), and DO (%) were monitored at 24-h intervals for a total duration of 120–140 h. The ethanol concentration was quantified via high-performance liquid chromatography (HPLC) with a refractive index detector.

## Supplementary Information

Below is the link to the electronic supplementary material.

Supplementary Material 1

## Data Availability

The raw sequencing data have been deposited in the NCBI Sequence Read Archive (SRA) under BioProject PRJNA1288782 (https://www.ncbi.nlm.nih.gov/bioproject/PRJNA1288782) with accession numbers SRX29599295–SRX29599302. The draft genome sequence of *M. gelida* PAMC 26583 (GCA_977009815) has been deposited in the European Nucleotide Archive (ENA) under BioProject PRJEB100863. The draft genome sequence of *M. robertii* PAMC 26600 (GCA_977005135) and mitochondrial genome sequences ( *M. gelida* PAMC 26583: GCA_977005115; *M. robertii* PAMC 26600: GCA_977005125) have been deposited in the ENA under BioProject PRJEB100532. In addition, the assembled genome and mitochondrial genome sequences, together with protein sequences, genome annotations, and orthologous gene group data for *M. gelida* PAMC 26583 and *M. robertii* PAMC 26600, are available in Figshare [57] ( https://doi.org/10.6084/m9.figshare.29500520.v1 ).
