# Supplementary material for: Genomic resources of Cold-adapted Mrakia yeasts and their potential biotechnological applications
Source: Sci Rep. 2025 Dec 22;15:45032. doi: 10.1038/s41598-025-29037-8 (PMC12749792; doi:10.1038/s41598-025-29037-8)
Supplement: Supplementary file 1 — Supplementary Material 1 [file 41598_2025_29037_MOESM1_ESM.docx]

# Supplementary Material for

# **Genomic Resources of Cold-Adapted *Mrakia* Yeasts and Their Potential Biotechnological Applications**

Seung Chul Shin^1^*, Dieu Linh Nguyen^1,2,3^, ChanSu Jeong^1,2^, Yung Mi Lee^1^, Jun Hyuck Lee^1,2^, Se Jong Han^1,2^, Han-Woo Kim^1,2^

^1^Division of Life Sciences, Korea Polar Research Institute (KOPRI), Incheon 21990, Republic of Korea

^2^Department of Polar Sciences, University of Science and Technology, Incheon 21990, Republic of Korea

^3^Current address: Department of Pathology and Genomic Medicine, Houston Methodist Hospital, Houston, Texas, USA

E-mail:S.C.S, [biotech21@gmail.com](mailto:biotech21@gmail.com); D.L.N, nudilinh@gmail.com; C.S.J., [wjdckstn3522@kopri.re.kr](mailto:wjdckstn3522@kopri.re.kr); Y.M.L, ymlee@kopri.re.kr; J.H.L, junhyucklee@kopri.re.kr; S.J.H, [hansj@kopri.re.kr](mailto:hansj@kopri.re.kr); H.W.K, [hwkim@kopri.re.kr](mailto:hwkim@kopri.re.kr)

*Correspondence: biotech21@gmail.com (S.C.S)


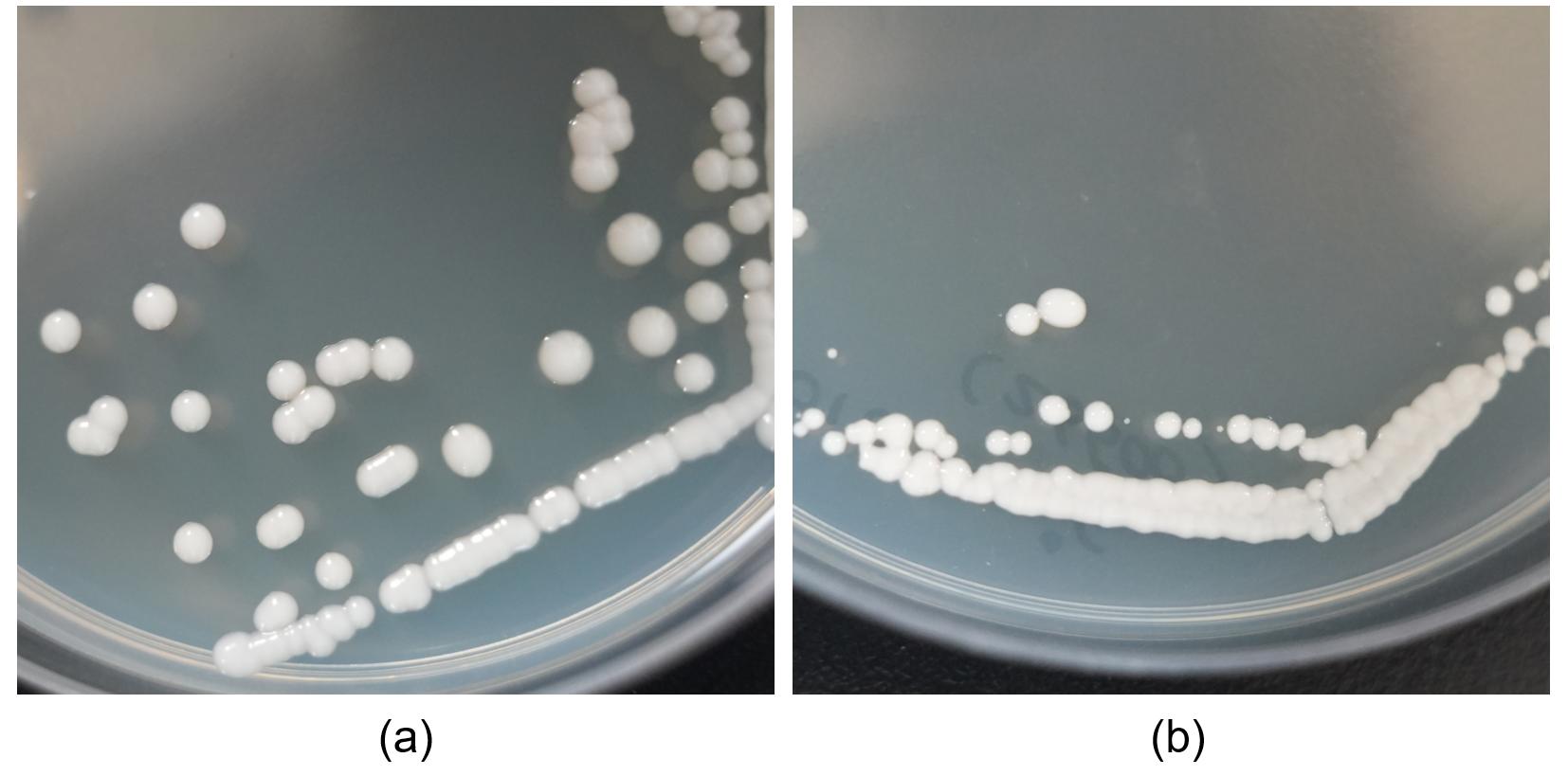


**Figure S1.** Colony morphology of PAMC 26583 (a) and PAMC 26600 (b) grown on MY agar at 10 °C for 10 days. Colonies on MY agar were circular, convex, entire, and glistening in both strains.


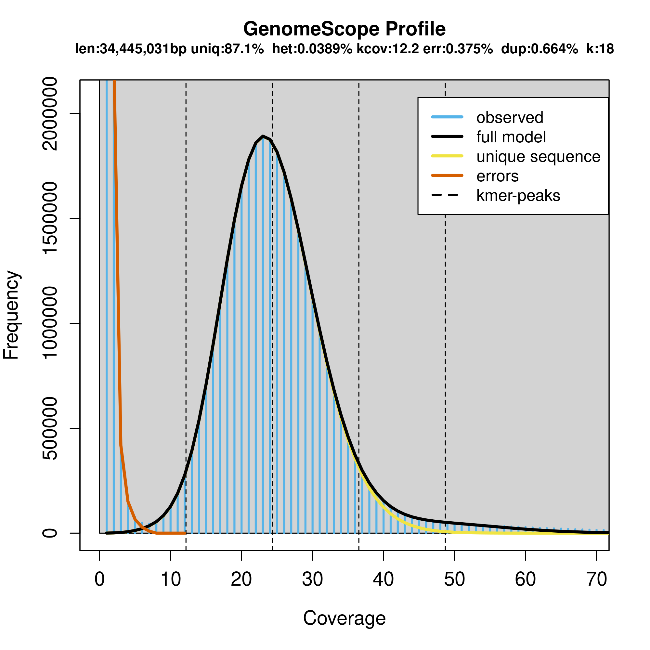

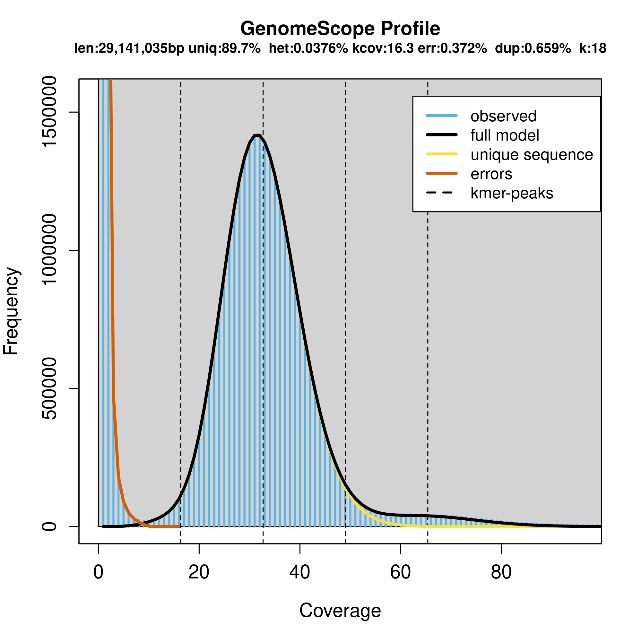


1. (b)

**Figure S2.** Genome size estimation of PAMC 26583 (a) and PAMC 26600 (b). K-mers (18 bp) were counted using Jellyfish, and genome sizes were predicted with GenomeScope. The estimated genome sizes for PAMC 26583 and PAMC 26600 were 34.45 Mb and 29.14 Mb, respectively


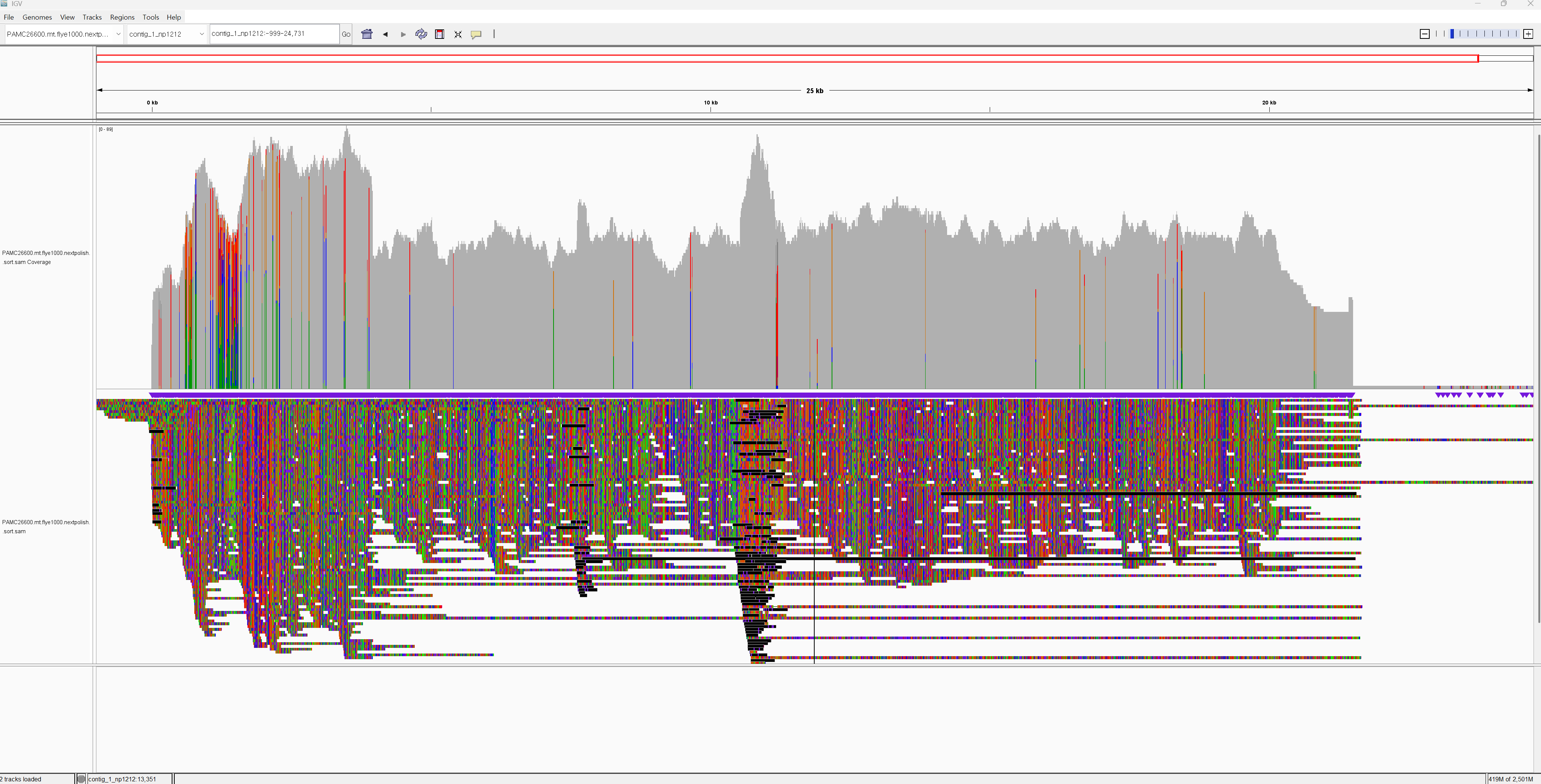


(a)


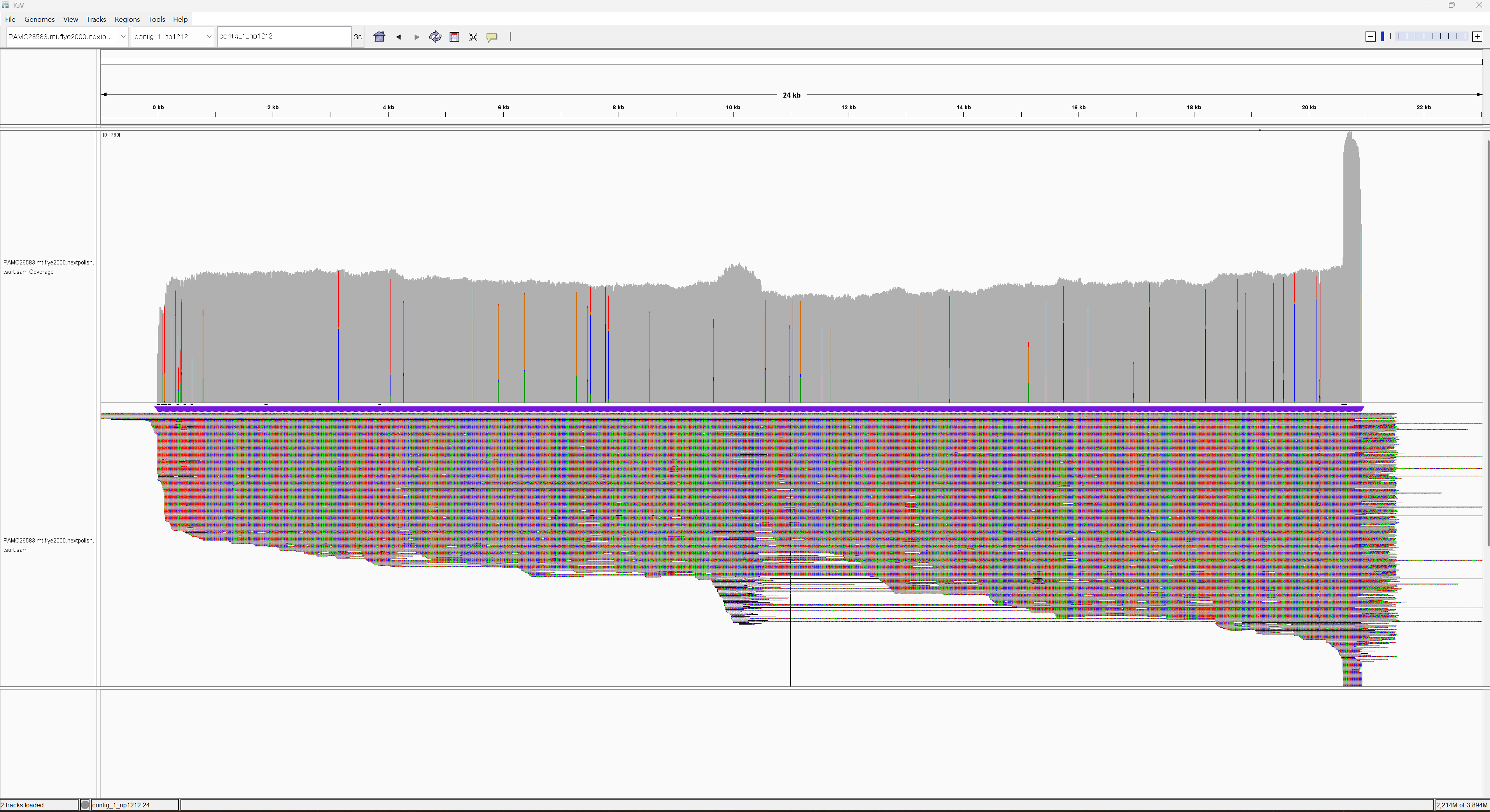


(b)

**Figure S3.** Alignment of Nanopore reads to the assembled mitochondrial genomes of *Mrakia* strains PAMC 26583 and PAMC 26600. Visualization of long-read alignments to the mitochondrial genome assemblies using IGV (Integrative Genomics Viewer, v2.16.1) to validate assembly quality. (a) Alignment of Nanopore reads to the mitochondrial genome of PAMC 26583. (b) Alignment of Nanopore reads to the mitochondrial genome of PAMC 26600. The consistent and dense coverage across most regions, along with the uniform read mapping, supports the accuracy and completeness of the mitochondrial genome assemblies in both strains.

**
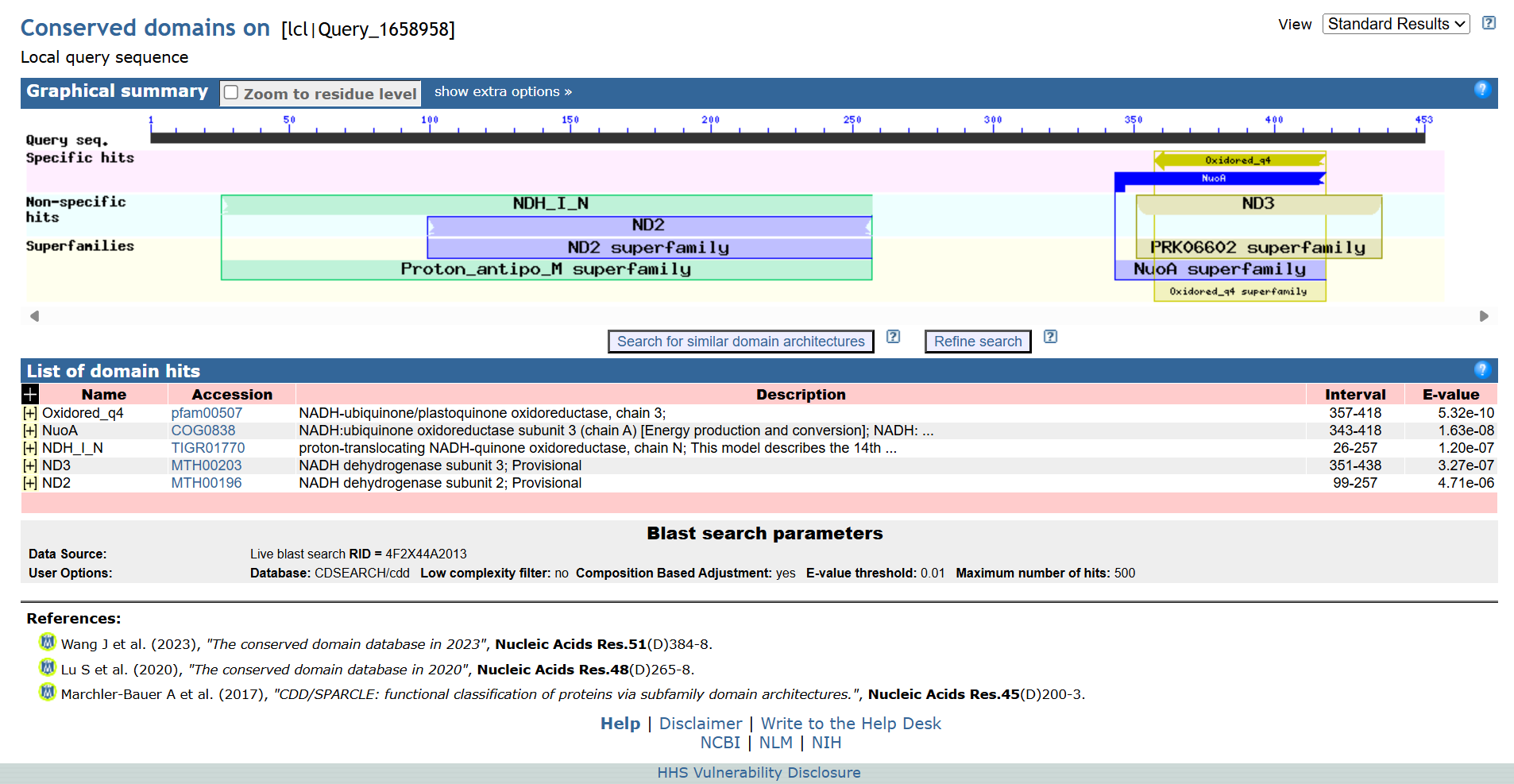
**

**Figure S4.** Conserved domain analysis of the putative ND2–ND3 fusion protein in the *Mrakia* mitochondrial genome. To investigate the unusual gene structure observed in the mitochondrial genome of *Mrakia* strains PAMC 26583 and PAMC 26600—where ND2 and ND3 appear as a single, fused open reading frame—we conducted a conserved domain search using BLASTP against the NCBI Conserved Domain Database (CDD). The query sequence corresponds to the predicted fusion protein. Domain hits ND2 and ND3, suggesting that both ND2 and ND3 regions are retained and conserved within the same polypeptide. These results support the hypothesis that ND2 and ND3 are translated as a single fusion protein in these strains.

**
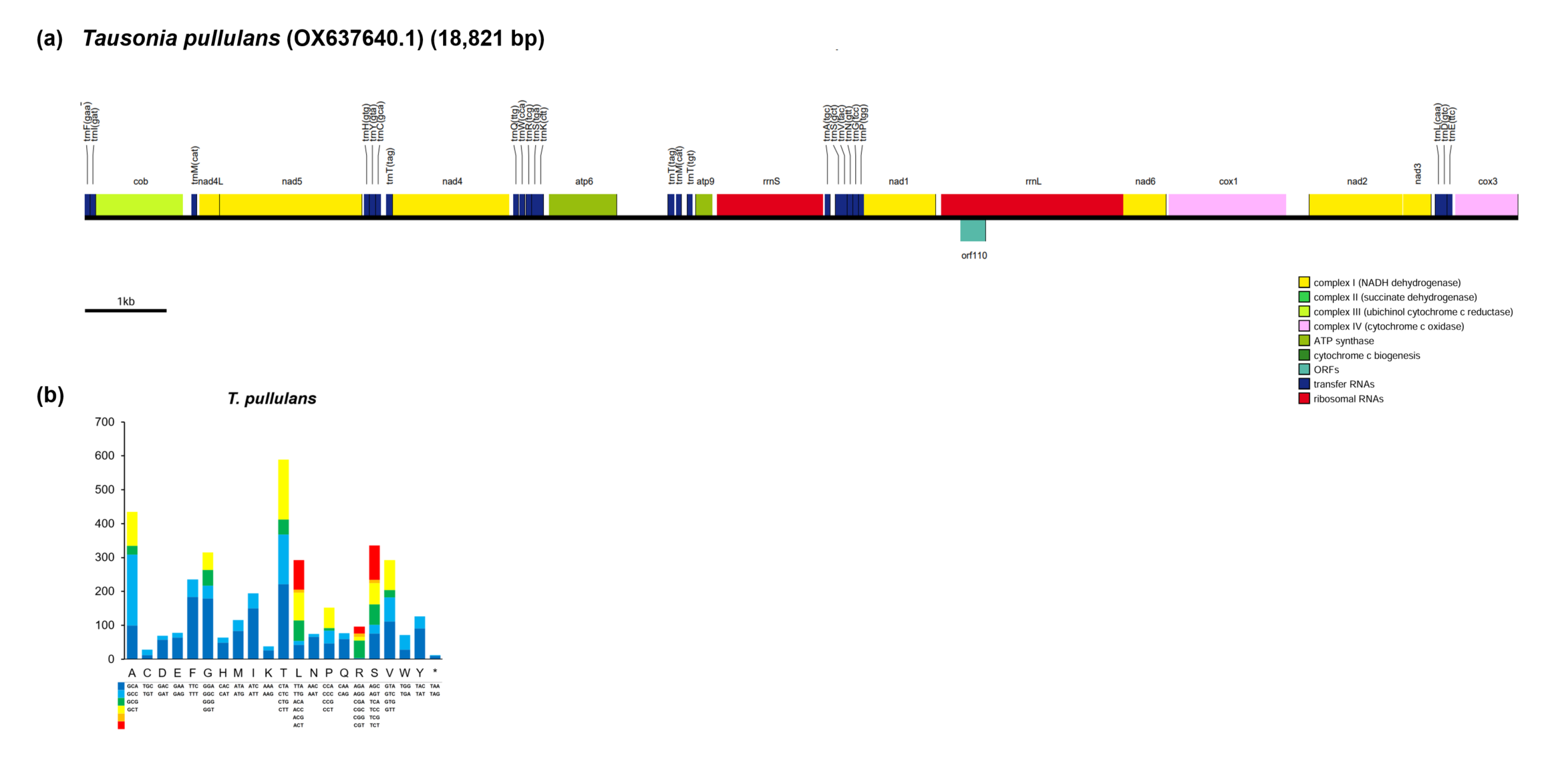
**

**Figure S5.** Mitogenome of *Tausonia pullulans* (OX637640.1). (a) The mitogenome is 18,821 bp and contains 11 protein-coding genes, 2 rRNA genes, and 24 tRNA genes. The types of genes are represented by boxes of different colors (legend in captions). It was not identified whether the mitogenome of this strain is linear or not. (b) codon usage in the mitogenome of *T. pullulans*. Frequency of codon usage is plotted on the y-axis.

**Table S1.** Sequencing Statistics for two yeast strains

|  |  | Sequencing platform | Instrument | Total reads | Total read base (bp) | Max length | GC (%) |
| --- | --- | --- | --- | --- | --- | --- | --- |
| PAMC 26583 | DNA | Nanopore read | PromethION | 1,111,469 | 3,923,634,917 | 74,376 | 74,376 |
|  |  | Illumina read | NovaSeq | 7,229,390 | 1,091,637,890 | 151 | 55.46 |
|  | RNA | Illumina read | NovaSeq | 91,875,892 | 9,279,465,092 | 101 | 56.19 |
|  |  |  |  |  |  |  |  |
| PAMC 26600 | DNA | Nanopore read | PromethION | 2,041,629 | 2,106,756,018 | 65,631 | 65,631 |
|  |  | Illumina read | NovaSeq | 8,231,262 | 1,242,920,562 | 151 | 55.08 |
|  | RNA | Illumina read | NovaSeq | 104,486,502 | 10,553,136,702 | 101 | 55.61 |

**Table S2.** *de novo* repeat sequence of PAMC 26583

|  | number of elements | length occupied (bp) | percentage of sequence (%) |
| --- | --- | --- | --- |
| **Retroelements** | **550** | **383,962** | **1.12** |
| SINEs: | 15 | 1,166 | 0 |
| Penelope: | 0 | 0 | 0 |
| LINEs: | 98 | 71,251 | 0.21 |
| CRE/SLACS | 31 | 21,005 | 0.06 |
| L2/CR1/Rex | 18 | 5,890 | 0.02 |
| R1/LOA/Jockey | 0 | 0 | 0 |
| R2/R4/NeSL | 0 | 0 | 0 |
| RTE/Bov-B | 0 | 0 | 0 |
| L1/CIN4 | 0 | 0 | 0 |
| LTR elements: | 437 | 311,545 | 0.91 |
| BEL/Pao | 18 | 19,472 | 0.06 |
| Ty1/Copia | 383 | 283,930 | 0.83 |
| Gypsy/DIRS1 | 36 | 8,143 | 0.02 |
| Retroviral | 0 | 0 | 0 |
|  |  |  |  |
| **DNA transposons** | **76** | **50,455** | **0.15** |
| hobo-Activator | 17 | 5,973 | 0.02 |
| Tc1-IS630-Pogo | 12 | 3,718 | 0.01 |
| En-Spm | 0 | 0 | 0 |
| MULE-MuDR | 47 | 40,764 | 0.12 |
| PiggyBac | 0 | 0 | 0 |
| Tourist/Harbinger | 0 | 0 | 0 |
| Other (Mirage, P-element, Transib) | 0 | 0 | 0 |
|  |  |  |  |
|  |  |  |  |
| **Rolling-circles** | **75** | **98,374** | **0.29** |
|  |  |  |  |
| **Unclassified:** | **3,675** | **1,206,346** | **3.53** |
|  |  |  |  |
| **Total interspersed repeats:** |  | **1,640,763** | **4.8** |
|  |  |  |  |
|  |  |  |  |
| **Small RNA:** | **15** | **1,166** | **0** |
|  |  |  |  |
| **Satellites:** | **0** | **0** | **0** |
| **Simple repeats:** | **27,986** | **1,287,273** | **3.76** |
| **Low complexity:** | **11,934** | **680,180** | **1.99** |
|  |  |  |  |
| **Total repeat sequence** |  | **3,706,590** | **10.83** |

**Table S3.** *de novo* repeat sequence of PAMC 26600

|  | number of elements | length occupied (bp) | percentage of sequence (%) |
| --- | --- | --- | --- |
| **Retroelements** | **550** | **383,962** | **1.12** |
| SINEs: | 15 | 1,166 | 0 |
| Penelope: | 0 | 0 | 0 |
| LINEs: | 98 | 71,251 | 0.21 |
| CRE/SLACS | 31 | 21,005 | 0.06 |
| L2/CR1/Rex | 18 | 5,890 | 0.02 |
| R1/LOA/Jockey | 0 | 0 | 0 |
| R2/R4/NeSL | 0 | 0 | 0 |
| RTE/Bov-B | 0 | 0 | 0 |
| L1/CIN4 | 0 | 0 | 0 |
| LTR elements: | 437 | 311,545 | 0.91 |
| BEL/Pao | 18 | 19,472 | 0.06 |
| Ty1/Copia | 383 | 283,930 | 0.83 |
| Gypsy/DIRS1 | 36 | 8,143 | 0.02 |
| Retroviral | 0 | 0 | 0 |
|  |  |  |  |
| **DNA transposons** | **76** | **50,455** | **0.15** |
| hobo-Activator | 17 | 5,973 | 0.02 |
| Tc1-IS630-Pogo | 12 | 3,718 | 0.01 |
| En-Spm | 0 | 0 | 0 |
| MULE-MuDR | 47 | 40,764 | 0.12 |
| PiggyBac | 0 | 0 | 0 |
| Tourist/Harbinger | 0 | 0 | 0 |
| Other (Mirage, P-element, Transib) | 0 | 0 | 0 |
|  |  |  |  |
|  |  |  |  |
| **Rolling-circles** | **75** | **98,374** | **0.29** |
|  |  |  |  |
| **Unclassified:** | **3,675** | **1,206,346** | **3.53** |
|  |  |  |  |
| **Total interspersed repeats:** |  | **1,640,763** | **4.8** |
|  |  |  |  |
|  |  |  |  |
| **Small RNA:** | **15** | **1,166** | **0** |
|  |  |  |  |
| **Satellites:** | **0** | **0** | **0** |
| **Simple repeats:** | **27,986** | **1,287,273** | **3.76** |
| **Low complexity:** | **11,934** | **680,180** | **1.99** |
|  |  |  |  |
| **Total repeat sequence** |  | **2,194,812** | **7.57** |

**Table S4.** Statistics of genome sequences used in this study

| species | The number of  scaffolds | The number of  contigs | The number of  proteins | scf N50 (kilo-bases) | ctg N50 (kilo-bases) | Total scaffold length (bases) | GOLD project ID* | NCBI Accession number |
| --- | --- | --- | --- | --- | --- | --- | --- | --- |
| PAMC 26583 | 28 | 28 | 8,918 | 2,043 | 2,043 | 34,210,599 |  | GCA_977009815 |
| PAMC 26600 | 15 | 15 | 8,131 | 2,493 | 2,493 | 28,976,436 |  | GCA_977005135 |
| *Cryptococcus curvatus* ATCC 20509 | 16 | 145 | 8,661 | 2,510 | 280 | 19,908,169 | Gp0235748 | GCA_001712445.1 |
| *Fellomyces penicillatus* Phaff54-35 | 22 | 40 | 8,340 | 2,828 | 1,095 | 21,049,006 | Gp0110604 | currently not available |
| *Filobasidium floriforme* CBS 6241 | 42 | 42 | 8,319 | 938 | 938 | 27,469,088 | Gp0644958 | GCA_019201745.1 |
| *Dioszegia hungarica* PDD-24b-2 | 17 | 17 | 8,219 | 2,166 | 2,166 | 20,956,533 | currently not available | GCF_025882075.1 |
| *Kockovaella imperatae* NRRL Y-17943 | 38 | 38 | 7,393 | 1,071 | 1,071 | 17,465,713 | Gp0120011 | GCA_002102565.1 |
| *Ustilago maydis* 521 | 27 | 253 | 6,785 | 884 | 129 | 19,664,388 | Gp0000206 | GCA_000328475.2 |
| *Naganishia vishniacii* ANT03-052 | 38 | 77 | 6,711 | 1,081 | 687 | 19,679,091 | Gp0022517 | GCA_015708705.1 |
| *Pseudozyma antarctica* | 27 | 759 | 6,640 | 730 | 42 | 18,065,590 | Gp0645215 | GCA_900322835.1 |
| *Cryptococcus neoformans var neoformans* JEC21 | 14 | 37 | 6,475 | 1,439 | 1,077 | 19,051,922 | Gp0000508 | GCA_000091045.1 |
| *Phaffia rhodozyma* | 16 | 16 | 6,424 | 1,844 | 1,844 | 19,509,598 | currently not available | GCA_037127225.1 |

* A unique identifier used within the Genomes OnLine Database (GOLD), which is maintained by the Joint Genome Institute (JGI).

**Table S5.** CAZyme genes in two yeast strains

| CAZyme ID | PAMC 26583 Hit numbers | PAMC 26583 Gene ID | PAMC 26600 Hit numbers | PAMC 26600 Gene ID |
| --- | --- | --- | --- | --- |
| GH001 | 1 | g6846.t1 | 3 | g3241.t1;g6394.t1;g6394.t2 |
| GH003 | 7 | g10.t1;g2352.t1;g2423.t1;g293.t1;g5284.t1;g5532.t1;g8555.t1 | 8 | g2925.t1;g3396.t1;g3624.t1;g3624.t2;g4311.t1;g4852.t1  ;g5332.t1;g7482.t1 |
| GH005 | 8 | g128.t1;g1663.t1;g5232.t1;g543.t1;g6747.t1;g7017.t1;g7017.t2;g8065.t1 | 10 | g1923.t1;g3429.t1;g3429.t2;g3429.t3;g4050.t1;g5003.t1  ;g5197.t1;g5452.t1;g6894.t1;g7890.t1 |
| GH009 | 1 | g6429.t1 | 1 | g2752.t1 |
| GH012 | 0 | None | 1 | g6478.t1 |
| GH013 | 20 | g2238.t1;g2238.t1;g2428.t1;g2807.t1;g2807.t1;g2807.t2  ;g2807.t2;g2829.t1;g4081.t1;g4081.t1;g4117.t1;g4966.t1  ;g4966.t2;g6320.t1;g7222.t1;g7986.t1;g8110.t1;g8169.t1  ;g8169.t1;g8461.t1 | 19 | g1293.t1;g1644.t1;g1644.t2;g2057.t1;g33.t1;g33.t1;g3484.t1  ;g4102.t1;g4196.t1;g4196.t1;g6210.t1;g6210.t1;g6357.t1  ;g6513.t1;g6513.t2;g6572.t1;g6572.t1;g7407.t1;g7574.t1 |
| GH015 | 3 | g5046.t1;g6695.t1;g6695.t2 | 1 | g6698.t1 |
| GH016 | 14 | g1339.t1;g1457.t1;g3070.t1;g3261.t1;g3492.t1;g3494.t1  ;g3494.t3;g445.t1;g4770.t1;g5525.t1;g6899.t1;g7215.t1;g7654.t1;g8.t1 | 16 | g1108.t1;g1108.t2;g1110.t1;g3194.t1;g3194.t2;g3478.t1  ;g4417.t1;g4659.t1;g4953.t1;g5097.t1;g5410.t1;g6875.t1  ;g7122.t1;g7356.t1;g804.t1;g922.t1 |
| GH017 | 1 | g8655.t1 | 1 | g7593.t1 |
| GH018 | 2 | g7509.t1;g8903.t1 | 4 | g4538.t1;g4679.t1;g4679.t2;g7802.t1 |
| GH020 | 1 | g4605.t1 | 1 | g5660.t1 |
| GH027 | 1 | g8116.t1 | 1 | g4111.t1 |
| GH028 | 4 | g3050.t1;g3050.t2;g870.t1;g870.t2 | 3 | g5016.t1;g546.t1;g546.t2 |
| GH029 | 3 | g7705.t1;g7705.t2;g7705.t3 | 1 | g4328.t1 |
| GH031 | 6 | g2234.t1;g4536.t1;g4635.t1;g7757.t1;g8486.t1;g8486.t2 | 3 | g4353.t1;g5641.t1;g7434.t1 |
| GH032 | 1 | g539.t1 | 1 | g5455.t1 |
| GH035 | 1 | g2027.t1 | 1 | g6048.t1 |
| GH037 | 2 | g280.t1;g7731.t1 | 3 | g1300.t1;g4338.t1;g4816.t1 |
| GH038 | 1 | g552.t1 | 0 | None |
| GH043 | 0 | None | 2 | g3281.t1;g617.t1 |
| GH047 | 2 | g3594.t1;g5113.t1 | 2 | g1200.t1;g6758.t1 |
| GH051 | 0 | None | 1 | g6829.t1 |
| GH063 | 2 | g1162.t1;g8218.t1 | 2 | g4210.t1;g761.t1 |
| GH071 | 4 | g4663.t1;g5817.t1;g6271.t1;g6271.t2 | 3 | g2129.t1;g2129.t2;g5712.t1 |
| GH072 | 1 | g1447.t1 | 0 | None |
| GH078 | 1 | g5773.t1 | 1 | g2581.t1 |
| GH079 | 5 | g210.t1;g2893.t1;g2893.t2;g3048.t1;g3427.t1 | 5 | g1047.t1;g2022.t1;g5018.t1;g6594.t1;g6595.t1 |
| GH092 | 3 | g5341.t1;g5341.t2;g5341.t3 | 1 | g7038.t1 |
| GH105 | 2 | g4544.t1;g8792.t1 | 1 | g5578.t1 |
| GH109 | 2 | g2093.t1;g685.t1 | 2 | g5351.t1;g6102.t1 |
| GH125 | 1 | g7272.t1 | 2 | g3556.t1;g3556.t2 |
| GH127 | 1 | g8862.t1 | 0 | None |
| GH128 | 5 | g2543.t1;g4265.t1;g5185.t1;g5185.t2;g6314.t1 | 5 | g1393.t1;g1394.t1;g1394.t2;g2053.t1;g6826.t1 |
| GH152 | 0 | None | 1 | g6681.t1 |
| PL001 | 1 | g836.t1 | 1 | g533.t1 |
| PL014 | 4 | g2320.t1;g2320.t2;g5227.t1;g6886.t1 | 2 | g3184.t1;g6898.t1 |
| PL035 | 2 | g2366.t1;g7755.t1 | 2 | g4350.t1;g6380.t1 |
| PL042 | 1 | g7197.t1 | 2 | g3518.t1;g3518.t2 |
| sum | 114 |  | 113 |  |

**Table S6.** Peptidase genes in two yeast strains

| MEROPS peptidase ID | PAMC 26583 Hit numbers | PAMC 26583 Gene ID | PAMC 26600 Hit numbers | PAMC 26600 Gene ID |
| --- | --- | --- | --- | --- |
| A01A | 3 | g2015.t1;g4601.t1;g4607.t1 | 2 | g5662.t1;g5662.t2 |
| C01B | 1 | g6368.t1 | 1 | g2085.t1 |
| C12 | 1 | g3027.t1 | 1 | g4974.t1 |
| C19 | 0 | None | 1 | g5567.t1 |
| I87 | 1 | g5189.t1 | 1 | g6822.t1 |
| M16B | 1 | g8894.t1 | 0 | None |
| M17 | 1 | g7175.t1 | 1 | g3496.t1 |
| M18 | 1 | g583.t1 | 0 | None |
| M19 | 1 | g7750.t1 | 1 | g4344.t1 |
| M20A | 5 | g3113.t1;g3113.t2;g5693.t1;g6237.t1;g1171.t1 | 4 | g2143.t1;g2715.t1;g5066.t1;g770.t1 |
| M20F | 1 | g7058.t1 | 1 | g3469.t1 |
| M24A | 0 | None | 1 | g6383.t1 |
| M38 | 1 | g3500.t1 | 3 | g6028.t1;g6029.t1;g1086.t1 |
| M43B | 1 | g2322.t1 | 1 | g6289.t1 |
| M67A | 1 | g5632.t1 | 1 | g2724.t1 |
| S09C | 0 | None | 2 | g397.t1;g398.t1 |
| S09X | 2 | g7493.t1;g8066.t1 | 2 | g4048.t1;g4518.t1 |
| S10 | 2 | g7111.t1;g137.t1 | 2 | g3418.t1;g1975.t1 |
| S28 | 2 | g8282.t1;g8282.t2 | 1 | g4251.t1 |
| S33 | 6 | g3834.t1;g7476.t1;g6718.t1;g5343.t1;g8325.t1;g8325.t2 | 4 | g7040.t1;g4588.t1;g322.t1;g3098.t1 |
| T01A | 6 | g3248.t1;g2574.t1;g5156.t1;g4593.t1;g8032.t1;g8158.t1 | 6 | g3655.t1;g4082.t1;g4131.t1;g5572.t1;g6790.t1;g5220.t1 |
| T02 | 1 | g6043.t1 | 1 | g2312.t1 |
| sum | 38 |  | 37 |  |

**Table S7.** Orthologous group related to potential cold-adaptation mechanisms

| Description | orthologous gene cluster | *Cryptococcus_curvatus* | *Cryptococcus_neoformans* | *Dioszegia_hungarica* | *Fellomyces_penicillatus* | *Filobasidium_floriforme* | *Kockovaella_imperatae* | *Naganishia_vishniacii* | *Mrakia gelida* PAMC 26583 | *Mrakia robertii* PAMC 26600 | *Pseudozyma_antarctica* | *Ustilago_maydis* | *Phaffia rhodozyma* | PFAMs | Definition |
| --- | --- | --- | --- | --- | --- | --- | --- | --- | --- | --- | --- | --- | --- | --- | --- |
| Fatty acid desaturase | **cluster8579** | 0 | 0 | 0 | 0 | 0 | 0 | 0 | 1 | 1 | 0 | 0 | 0 | Cyt-b5,FA_desaturase | Delta 9 fatty acid desaturase |
|  | cluster2514 | 1 | 1 | 1 | 1 | 1 | 1 | 1 | 1 | 1 | 1 | 1 | 1 | Cyt-b5,FA_desaturase | Delta 9 fatty acid desaturase |
|  | **cluster7962** | 0 | 0 | 0 | 0 | 0 | 0 | 0 | 1 | 1 | 0 | 0 | 1 | DUF3474,FA_desaturase | Delta 12 fatty acid desaturase |
|  | cluster405 | 1 | 1 | 2 | 1 | 1 | 1 | 1 | 1 | 1 | 1 | 1 | 1 | DUF3474,FA_desaturase | Delta 12 fatty acid desaturase |
|  | **cluster8929** | 0 | 0 | 0 | 0 | 0 | 0 | 0 | 1 | 1 | 0 | 0 | 0 | DUF3474,FA_desaturase | Delta 12 fatty acid desaturase |
| Major Facilitator Superfamily | **cluster10086** | 0 | 0 | 0 | 0 | 0 | 0 | 0 | 1 | 1 | 0 | 0 | 0 | MFS_1 | Monocarboxylate transporter |
|  | **cluster10155** | 0 | 0 | 0 | 0 | 0 | 0 | 0 | 1 | 1 | 0 | 0 | 0 | MFS_1 | hypothetical protein QFC24_006696 |
|  | **cluster10849** | 0 | 0 | 0 | 0 | 0 | 0 | 0 | 1 | 1 | 0 | 0 | 0 | MFS_1 | major facilitator superfamily domain-containing protein |
|  | cluster116 | 2 | 1 | 1 | 1 | 2 | 2 | 1 | 2 | 1 | 1 | 1 | 1 | MFS_1,Sugar_tr | major facilitator superfamily domain-containing protein |
|  | cluster119 | 3 | 2 | 1 | 3 | 1 | 1 | 0 | 2 | 2 | 1 | 0 | 0 | MFS_1 | CNF01220-like protein |
|  | cluster14 | 2 | 3 | 2 | 2 | 2 | 2 | 1 | 4 | 2 | 1 | 1 | 1 | AA_permease_2,CBP,MFS_1,  Mpv17_PMP22 | Synaptic vesicle transporter SVOP and related transporters (major facilitator superfamily) |
|  | cluster1459 | 3 | 1 | 0 | 2 | 0 | 0 | 1 | 1 | 1 | 1 | 1 | 1 | MFS_1 | Permease of the major facilitator superfamily |
|  | cluster156 | 0 | 3 | 1 | 2 | 1 | 2 | 1 | 1 | 1 | 1 | 1 | 1 | MFS_1 | major facilitator superfamily domain-containing protein |
|  | cluster157 | 2 | 2 | 2 | 3 | 0 | 1 | 0 | 0 | 1 | 0 | 0 | 4 | MFS_1 | hypothetical protein NliqN6_3425 |
|  | cluster1814 | 1 | 1 | 1 | 1 | 1 | 1 | 1 | 1 | 1 | 1 | 1 | 1 | MFS_1,RRM_1 | Permease of the major facilitator superfamily |
|  | cluster196 | 1 | 3 | 1 | 1 | 2 | 0 | 1 | 1 | 1 | 1 | 1 | 1 | MFS_1,Pyr_redox_2,Sugar_tr | hypothetical protein C6P46_003869 |
|  | cluster218 | 2 | 1 | 1 | 1 | 1 | 1 | 2 | 1 | 1 | 1 | 1 | 1 | MFS_1 | Synaptic vesicle transporter SVOP and related transporters (major facilitator superfamily) |
|  | cluster276 | 3 | 1 | 1 | 1 | 1 | 1 | 0 | 1 | 1 | 1 | 1 | 1 | MFS_1 | Synaptic vesicle transporter SVOP and related transporters (major facilitator superfamily) |
|  | cluster2965 | 1 | 1 | 1 | 1 | 1 | 1 | 1 | 0 | 1 | 1 | 1 | 1 | MFS_1,Sugar_tr | Synaptic vesicle transporter SV2 (major facilitator superfamily) |
|  | cluster30 | 1 | 1 | 2 | 2 | 4 | 3 | 2 | 1 | 2 | 0 | 1 | 2 | MFS_1 | urease accessory protein |
|  | cluster301 | 1 | 2 | 1 | 1 | 1 | 1 | 1 | 2 | 2 | 0 | 0 | 1 | MFS_1 | MFS transporter |
|  | cluster3097 | 0 | 2 | 2 | 2 | 1 | 1 | 0 | 1 | 1 | 1 | 0 | 0 | MFS_1 | hypothetical protein FRC02_009347 |
|  | cluster3233 | 1 | 0 | 1 | 1 | 1 | 1 | 1 | 1 | 1 | 1 | 1 | 1 | MFS_1 | Synaptic vesicle transporter SVOP and related transporters (major facilitator superfamily) |
|  | cluster3404 | 1 | 0 | 1 | 1 | 0 | 1 | 0 | 2 | 2 | 1 | 1 | 0 | MFS_1,Sugar_tr | MFS general substrate transporter |
|  | cluster3406 | 0 | 0 | 0 | 0 | 1 | 0 | 0 | 3 | 2 | 1 | 1 | 2 | MFS_1 | fructose facilitator |
|  | cluster3449 | 1 | 1 | 1 | 1 | 1 | 1 | 1 | 1 | 1 | 0 | 0 | 1 | MFS_1,SBF,Sugar_tr | Synaptic vesicle transporter SVOP and related transporters (major facilitator superfamily) |
|  | cluster35 | 4 | 2 | 2 | 3 | 2 | 1 | 2 | 1 | 1 | 1 | 0 | 1 | MFS_1 | hypothetical protein NCC49_005136 |
|  | cluster352 | 2 | 1 | 1 | 1 | 1 | 1 | 1 | 1 | 1 | 1 | 1 | 1 | Fn3-like,MFS_1,Sugar_tr | LOW QUALITY PROTEIN: MFS general substrate transporter |
|  | cluster3560 | 0 | 1 | 1 | 1 | 1 | 1 | 1 | 1 | 1 | 1 | 0 | 1 | AMP-binding,AMP-binding_C,MFS_1,Sugar_tr,  Wbp11 | Permease of the major facilitator superfamily |
|  | cluster4041 | 0 | 0 | 2 | 1 | 1 | 1 | 1 | 1 | 1 | 0 | 0 | 1 | MFS_1 | Permease of the major facilitator superfamily |
|  | cluster4139 | 1 | 0 | 0 | 1 | 0 | 1 | 1 | 1 | 1 | 1 | 1 | 1 | MFS_1 | Monocarboxylate transporter |
|  | cluster4169 | 1 | 2 | 0 | 0 | 0 | 0 | 1 | 2 | 2 | 0 | 0 | 1 | MFS_1 | uncharacterized protein CI109_000505 |
|  | cluster4214 | 0 | 1 | 1 | 1 | 1 | 0 | 0 | 2 | 2 | 0 | 0 | 1 | MFS_1 | Permease of the major facilitator superfamily |
|  | cluster4508 | 0 | 1 | 1 | 2 | 0 | 1 | 0 | 1 | 1 | 0 | 0 | 1 | MFS_1 | Predicted transporter (major facilitator superfamily) |
|  | cluster4689 | 1 | 1 | 0 | 1 | 1 | 1 | 0 | 1 | 1 | 0 | 0 | 0 | MFS_1 | major facilitator superfamily domain-containing protein |
|  | cluster4786 | 0 | 1 | 1 | 1 | 1 | 0 | 1 | 1 | 1 | 0 | 0 | 0 | MFS_1 | allantoate permease |
|  | cluster4797 | 1 | 1 | 1 | 0 | 1 | 0 | 1 | 1 | 1 | 0 | 0 | 0 | MFS_1 | major facilitator superfamily domain-containing protein |
|  | cluster483 | 1 | 3 | 2 | 1 | 1 | 1 | 1 | 1 | 2 | 0 | 0 | 0 | MFS_1 | major facilitator superfamily domain-containing protein |
|  | cluster5082 | 0 | 0 | 0 | 0 | 1 | 0 | 2 | 1 | 1 | 1 | 1 | 0 | MFS_1 | MFS transporter, NNP family, nitrate/nitrite transporter, partial |
|  | cluster5505 | 2 | 0 | 0 | 0 | 1 | 0 | 0 | 1 | 1 | 0 | 0 | 0 | MFS_1 | hypothetical protein CBS101457_003117 |
|  | cluster5614 | 1 | 0 | 0 | 0 | 1 | 0 | 1 | 1 | 1 | 0 | 0 | 0 | Glyco_hydro_61,MFS_1 | hypothetical protein |
|  | cluster5778 | 1 | 1 | 1 | 0 | 0 | 0 | 0 | 1 | 1 | 0 | 0 | 0 | MFS_1 | Monocarboxylate transporter |
|  | cluster5988 | 0 | 1 | 0 | 0 | 0 | 0 | 0 | 2 | 2 | 0 | 0 | 0 | MFS_1 | uncharacterized protein CI109_000505 |
|  | cluster6049 | 0 | 0 | 1 | 0 | 0 | 0 | 0 | 1 | 1 | 0 | 0 | 1 | MFS_1 | Permease of the major facilitator superfamily |
|  | cluster62 | 1 | 3 | 1 | 2 | 1 | 1 | 2 | 2 | 3 | 0 | 0 | 2 | MFS_1 | Permease of the major facilitator superfamily |
|  | cluster6495 | 0 | 0 | 1 | 0 | 1 | 0 | 0 | 0 | 1 | 0 | 0 | 1 | MFS_1 | hypothetical protein FFLO_01168 |
|  | **cluster7390** | 0 | 0 | 0 | 0 | 0 | 0 | 0 | 2 | 1 | 0 | 0 | 0 | DUF3632,MFS_1 | Permease of the major facilitator superfamily |
|  | **cluster8230** | 0 | 0 | 0 | 0 | 0 | 0 | 0 | 1 | 1 | 0 | 0 | 0 | DUF3632,MFS_1 | Permease of the major facilitator superfamily |
|  | **cluster8340** | 0 | 0 | 0 | 0 | 0 | 0 | 0 | 1 | 1 | 0 | 0 | 0 | MFS_1 | hypothetical protein B0H11DRAFT_2276462 |
|  | **cluster8592** | 0 | 0 | 0 | 0 | 0 | 0 | 0 | 1 | 1 | 0 | 0 | 0 | MFS_1,Sugar_tr | hypothetical protein B9479_005650 |
|  | **cluster9736** | 0 | 0 | 0 | 0 | 0 | 0 | 0 | 1 | 1 | 0 | 0 | 0 | MFS_1 | Synaptic vesicle transporter SVOP and related transporters (major facilitator superfamily) |
|  | **cluster9955** | 0 | 0 | 0 | 0 | 0 | 0 | 0 | 1 | 1 | 0 | 0 | 0 | MFS_1 | hypothetical protein B0H10DRAFT_2094012 |
| superoxide dismutase | **cluster8223** | 0 | 0 | 0 | 0 | 0 | 0 | 0 | 1 | 1 | 0 | 0 | 0 | Sod_Cu | Cu,Zn superoxide dismutase-like protein |
|  | **cluster7505** | 0 | 0 | 0 | 0 | 0 | 0 | 0 | 1 | 1 | 0 | 0 | 1 | Sod_Fe_C | Manganese/iron superoxide dismutase, C-terminal |
|  | **cluster6841** | 0 | 0 | 0 | 0 | 0 | 0 | 0 | 1 | 1 | 0 | 0 | 1 | Hydrolase_like,Sod_Fe_C,  Sod_Fe_N | manganese superoxide dismutase |
|  | cluster223 | 1 | 1 | 1 | 1 | 1 | 1 | 1 | 1 | 1 | 2 | 2 | 1 | Hydrolase_like,Sod_Fe_C,  Sod_Fe_N | manganese superoxide dismutase |
| catalase | cluster3989 | 2 | 1 | 1 | 1 | 1 | 0 | 1 | 1 | 1 | 0 | 0 | 1 | Catalase,Catalase-rel | catalase |
|  | cluster4331 | 0 | 2 | 1 | 0 | 1 | 1 | 1 | 1 | 1 | 0 | 0 | 1 | Catalase,Catalase-rel,DJ-1_PfpI,TRM | catalase |
| glutathione peroxidase | cluster491 | 1 | 1 | 1 | 1 | 1 | 1 | 1 | 1 | 2 | 1 | 1 | 1 | GSHPx | glutathione peroxidase |
|  | cluster4229 | 1 | 1 | 1 | 1 | 0 | 1 | 1 | 1 | 1 | 0 | 0 | 1 | GSHPx | glutathione peroxidase |
| trehalose synthase | cluster694 | 1 | 1 | 1 | 1 | 1 | 1 | 1 | 1 | 1 | 1 | 1 | 1 | Glyco_transf_20,  Trehalose_PPase | trehalose 6-phosphate phosphatase, partial |
| trehalose synthase | cluster468 | 1 | 2 | 1 | 1 | 1 | 1 | 1 | 1 | 1 | 1 | 1 | 1 | Glyco_transf_20,  Trehalose_PPase | trehalose 6-p synthase |
